# Transcriptional profiling of dental sensory and proprioceptive trigeminal neurons using single-cell RNA sequencing

**DOI:** 10.1038/s41368-023-00246-z

**Published:** 2023-09-25

**Authors:** Pa Reum Lee, Jihoon Kim, Heather Lynn Rossi, Sena Chung, Seung Yub Han, Junhyong Kim, Seog Bae Oh

**Affiliations:** 1 Department of Neurobiology and Physiology, School of Dentistry and Dental Research Institute, Seoul National University, Seoul, Republic of Korea; 2 Genomics and Computational Biology Graduate Group, University of Pennsylvania, Philadelphia, PA USA; 3 Department of Pathobiology, University of Pennsylvania, Philadelphia, PA USA; 4 Department of Biology, University of Pennsylvania, Philadelphia, PA USA; 5 Present Address: Brain Science Institute, Korea Institute of Science and Technology (KIST), Seoul, Republic of Korea

**Keywords:** Bioinformatics, Mechanisms of disease, RNA sequencing

## Abstract

Dental primary afferent (DPA) neurons and proprioceptive mesencephalic trigeminal nucleus (MTN) neurons, located in the trigeminal ganglion and the brainstem, respectively, are essential for controlling masticatory functions. Despite extensive transcriptomic studies on various somatosensory neurons, there is still a lack of knowledge about the molecular identities of these populations due to technical challenges in their circuit-validated isolation. Here, we employed high-depth single-cell RNA sequencing (scRNA-seq) in combination with retrograde tracing in mice to identify intrinsic transcriptional features of DPA and MTN neurons. Our transcriptome analysis revealed five major types of DPA neurons with cell type-specific gene enrichment, some of which exhibit unique mechano-nociceptive properties capable of transmitting nociception in response to innocuous mechanical stimuli in the teeth. Furthermore, we discovered cellular heterogeneity within MTN neurons that potentially contribute to their responsiveness to mechanical stretch in the masseter muscle spindles. Additionally, DPA and MTN neurons represented sensory compartments with distinct molecular profiles characterized by various ion channels, receptors, neuropeptides, and mechanoreceptors. Together, our study provides new biological insights regarding the highly specialized mechanosensory functions of DPA and MTN neurons in pain and proprioception.

## Introduction

Eating is an evolutionarily conserved behavior that requires a constant input of sensory information from the teeth and oral cavity, as well as proprioceptive feedback to the central nervous system (CNS) for coordinating chewing.^1,2]^ Healthy teeth are equipped with specific sensory nerve fibers that respond to mechanical stimuli such as food texture, vibration, and pressure.^1,2^ However, in the event of tooth damage, these fibers can transmit pain in response to normally innocuous stimuli, including an air puff or a water spray, to alert the organism of the damage before it affects the tooth’s survival.^3^ Additionally, muscle spindles within the jaw muscles convey stretch and proprioceptive information from the jaw muscles by monitoring changes in muscle length during the rhythmic movements of chewing.^4^

Two distinct pseudo-unipolar sensory afferents of the trigeminal nerve relay sensory information from the teeth and jaw-closing muscle spindles.^5^ Dental primary afferent (DPA) neurons innervate the teeth and have cell bodies located in the trigeminal ganglion (TG), a part of the peripheral nervous system (PNS), along with dorsal root ganglion (DRG) neurons.^5,6^ By contrast, the cell bodies of proprioceptive sensory afferents from the jaw-closing muscle spindles reside in the mesencephalic trigeminal nucleus (MTN) located within the brainstem of the CNS.^4,7^ Nguyen and colleagues were pioneers in characterizing the transcriptional identity of TG neurons using single-cell RNA sequencing (scRNA-seq) where they found 13 unique clusters.^8^ These identified clusters notably differed from DRG neurons, presumably due to the absence of proprioceptive neurons in the TG, as well as a subset of TG neurons innervating unique environments, such as the oral cavity and facial organs.^8^ Although a recent study has classified DPA neurons using fluorescence in situ hybridization (FISH) with multiple probes in mice,^9^ comprehensive transcriptomic profiling has not yet been performed on specific subsets of trigeminal neurons, including DPA neurons and proprioceptive MTN neurons. Given that DPA neurons primarily serve nociceptive functions in response to diverse noxious and even innocuous stimuli,^3,5^ while MTN neurons exclusively handle mechanoreceptive functions for proprioception,^4^ transcriptome profiling of these two distinct somatosensory neurons (DPA neurons *vs*. MTN neurons), differing in function and location, will help us to understand the nature of somatosensation (e.g., pain and proprioception) and their unique properties at the molecular and cellular level. It will also establish a baseline for future studies that will infer molecular changes in the face of injury.

In this study, we employed scRNA-seq to generate transcriptomic profiles of both DPA and MTN neurons, which were selected based on retrograde dyes from the maxillary first molar teeth and the masseter muscle, respectively. We applied scRNA-seq with high-depth coverage to individually isolated neurons^10^ to detect more genes with very low expression levels in individual cells and to identify cell type classification, cell type-specific enrichment, and molecular signatures in DPA and MTN neurons. Recently, rapid advances in scRNA-seq have facilitated the classification of various somatosensory neurons, such as those in the TG^8,11^ and DRG.^11–13^ More recently, high-throughput methods for single-nucleus RNA sequencing (snRNA-seq) have been employed to easily sort cells and prepare libraries from TG somatosensory neurons displaying heterogeneity in size and shape.^14,15^ Though these studies have provided valuable insights, it has been noted that the number of transcripts in the nucleus is considerably lower than that in the soma, suggesting that nuclear transcripts might not comprehensively capture the entire transcriptome profile of an individual cell.^16–18^ Moreover, this approach potentially excludes localized subcellular RNA transcripts that play essential roles in cellular functions.^16–18^ Therefore, scRNA-seq of individually isolated neurons stands as an appropriate approach for detecting biologically meaningful transcriptional changes from samples with low RNA inputs, owing to its higher sensitivity, larger number of detectable genes, and elimination of multiplets.^19^ Furthermore, manual isolation allows additional characterization such as cell morphology. We thus constructed the DPA and MTN transcriptome datasets to identify the transcriptional landscapes of two distinct sensory distinct sensory afferent constituents that are highly specialized in the trigeminal nerves.

## Results

### Performing scRNA-seq in DPA neurons innervating the tooth pulp

**Fig. 1:**
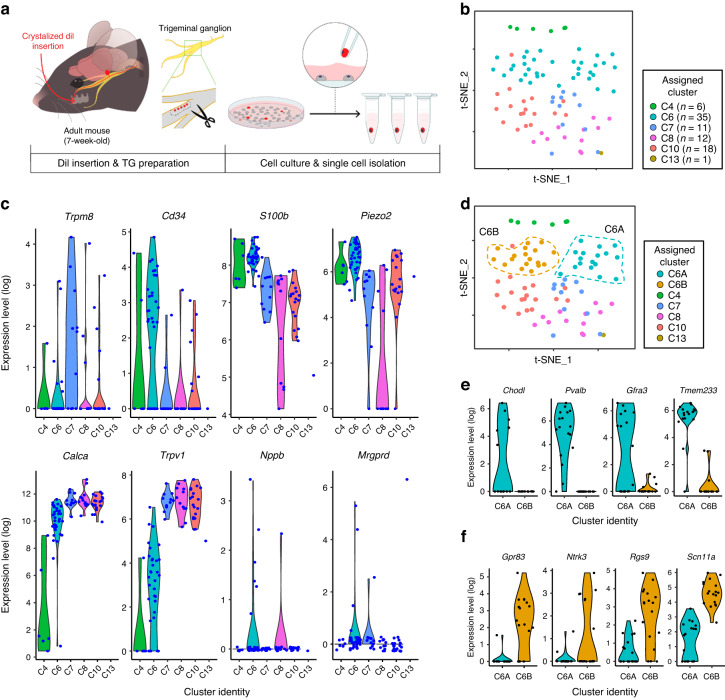
Single-cell RNA sequencing (scRNA-seq) identified dental primary afferent (DPA) neuron subtypes. **a** Schematic workflow of scRNA-seq for DPA neurons. **b** A t-distributed stochastic neighbor embedding (t-SNE) plot showing six distinct clusters, C4, C6, C7, C8, C10, and C13, identified in a total of 83 DPA neurons from 10 mice. The cluster labels are referenced from a previously published scRNA-seq dataset of the TG.^8^ Separated clusters are shown in different colors. **c** Violin plots showing expression of subtype-specific enriched genes [e.g., *Trpm8*, *Cd34*, *S100b*, *Piezo2*, *Calca*, *Trpv1*, *Nppb*, and *Mrgprd*, presented in TG^8^] in DPA neurons. **d** Two new sub-clusters exhibiting in C6, separated by C6A or C6B. **e**, **f** Most enriched genes in C6A [e.g., *Chodl*, *Pvalb*, *Gfra3*, and *Tmem233*] and C6B [e.g., *Gpr83*, *Ntrk3*, *Rgs9*, and *Scn11a*] (for more details, refer to Table S1)

We performed scRNA-seq on DiI-labeled DPA neurons manually collected from the culture of the TG using SMART-Seq protocol, as illustrated in Fig. 1a. To ensure the DPA neuron identity, we selected cell bodies of DPA neurons with DiI labeling in the TG, specifically innervating the maxillary first molars (Fig. 1a and Fig. S1a). Additionally, we confirmed that all DiI-labeled cells expressed Advillin, a marker for somatic sensory neurons^20^ (Fig. S1b).

We first assigned the uniquely mapped reads to the RefSeq annotated genes to generate gene counts (Fig. S1c), then normalized the dataset to mitigate differences in sequencing library depth.^21,22^ The transcriptomes of a total of 83 DPA neurons collected from 10 adult mice showed an average of 11 936 detected genes per neuron. We observed no significant cross-sample contamination (data not shown) and noted no noticeable correlation among the number of detected genes (Fig. S1d). The dataset also passed our quality control criteria (Fig. S1e, f) and underwent validation with reference genes, including housekeeping genes [e.g., *Gapdh* and *Actb* (β-actin)], sensory neuronal markers [e.g., *Tubb3* (β-tubulin III), *Uchl1* (PGP9.5), *Avil* (Advillin), and *Rbfox3* (NeuN)],^12,13^ glial markers [*Gfap* (Glial fibrillary acidic protein), *Kcnj10* (Kir4.1), and *Gja1* (Connexin-43)],^23^ and motor neuronal markers [e.g., *Chat* (ChAT) and *Neurog2* (Neurogenin-2)]^24^ (Fig. S1g).

### Identification of DPA neuron subtypes

Since it is generally accepted that somatosensory neurons are a heterogeneous cell population that encompasses touch (or low-threshold mechanosensitive)- and pain-related neurons, we leveraged a previously published scRNA-seq dataset of the TG,^8^ of which DPA neurons are a subset. This TG dataset used droplet-based sequencing on 3 500 neurons to identify and characterize 13 distinct classes of cells within the TG. Using Seurat’s TransferAnchor function,^25^ we assigned cell subtype cluster labels to our DPA neuron datasets based on previously established cluster identities, predicted to have the following functional classifications: C1-C2 cool-responsive neurons, C3 C-low threshold mechanoreceptors (C-LTMRs), C4-C6 Aβ-LTMRs/Aδ-LTMRs/Aδ-nociceptors respectively, C7-C10 different subtypes of polymodal peptidergic (PEP) nociceptors, C12 itch-transmitting neurons, and C13 non-peptidergic (NP) nociceptors.^8^ The 2-dimensional visualizations of well-separated clusters with different colors are displayed in Fig. 1b.

Our DPA neurons were annotated to six of the 13 TG clusters: namely C4, C6, C7, C8, C10, and C13, as per the cluster numbers of the TG dataset (Fig. 1b), according to the highest Seurat prediction score (Fig. S2a). Of note, since only a single DPA neuron out of 83 was annotated to C13 (Fig. 1b), we could not show its density plot in Fig. S2a. This single neuron assigned to C13 exhibited especially high expression of *Mrgprd*, which is a mas-related G protein-coupled receptor channel implicated in noxious mechanical pain transduction (Fig. 1c and Fig. S1h). This gene *Mrgprd* was also suggested as a marker gene for C13, predicted to be NP mechanosensitive nociceptors.^8^ The other 82 DPA neurons were annotated to five of the 13 previously defined TG clusters, with C6 (putative Aδ-nociceptors) being particularly abundant (42.2%, *n* = 35 of 83 neurons, as shown in Fig. 1b). In addition, these five DPA neuron clusters consisted of neurons with distinct cell body sizes (C7/C8/C10, predictive of polymodal PEP nociceptors appearing smaller than C6, predictive of Aδ-nociceptors on average) that supported further classification (Fig. S1i).

To compare our clusters with the clusters reported for the TG, we investigated the transcript levels of representative marker genes of the 13 TG clusters^8,9^ (Fig. 1c and Fig. S1h). These markers included a menthol receptor, *Trpm8* (C1-C2, cool-responsive); a cell surface antigen, *Cd34* (C3, C-LTMRs); a calcium-binding protein, *S100b* and a mechanosensitive ion channel, *Piezo2* (C4–C6, respectively Aβ-LTMRs/Aδ-LTMRs/Aδ-nociceptors); calcitonin gene-related peptide, *Calca*, a heat and capsaicin receptor, *Trpv1*, and a wasabi receptor, *Trpa1* (C7-C10, polymodal PEP nociceptors); natriuretic polypeptide B, *Nppb* (C11, itch-related neuropeptide); and *Mrgprd* (C13, NP nociceptors). While *Trpm8* and *Cd34* were highly expressed in C7 and C6 neurons respectively, the expression patterns of other representative genes, such as *S100b*, *Piezo2*, *Calca*, *Trpv1*, *Nppb*, and *Mrgprd*, were comparable with those of TG neurons in general (Fig. 1c and Fig. S1h). *Mrgpra3* (C12, itch related), another mas-related G protein-coupled receptor channel mediating chloroquine-evoked itch, was filtered out as it was expressed in fewer than three cells. Therefore, our findings indicate that retrograde tracer-labeled DPA neurons fall within a subset of all TG neurons but seem to exclude some cluster markers that are possibly more associated with oral sensory afferents from the gingiva, palate, or tongue, facial skin afferents, or other non-dental afferents.

We also observed that the C6 neurons, presumed to be Aδ-nociceptors,^8,9^ exhibited notable expression of both *Piezo2* and *Calca* (Fig. 1c), which could contribute to rapid nociception elicited by low-threshold mechanical stimulation. However, given the relatively lower expression of *Trpv1* in C6 compared to that in C7/C8/C10 (Fig. 1c), we inferred that C6 is more associated with Aβ-fibers than with Aδ-fibers. More specifically, the DPA neurons belonging to C6 were sub-divided into two new sub-clusters, namely C6A and C6B, based on distinct expression profiles (Fig. 1d). The C6A sub-cluster displayed significantly elevated expression of *Chodl* (Chondrolectin), *Pvalb* (Parvalbumin), *Gfra3* (GDNF family receptor alpha 3), and *Tmem233*, which encodes a transmembrane protein involved in the regulation of a voltage-gated sodium channel^26^ (Fig. 1e and Table S1), which suggested the C6A sub-cluster are Aβ-fiber slowly adapting (SA) LTMRs.^27^ The C6B sub-cluster showed enrichment of *Gpr83* (G protein-coupled receptor 83), *Ntrk3* (TRKC), *Rgs9* (Regulator of G protein signaling 9), and *Scn11a* (Nav1.9) (Fig. 1f and Table S1), suggesting its classification as Aβ-nociceptors.^27^ Together, these findings demonstrate that DPA neurons consist of five major types that correspond to C4, C6, C7, C8, and C10.

### Subtype-specific gene signatures of DPA neurons

**Fig. 2:**
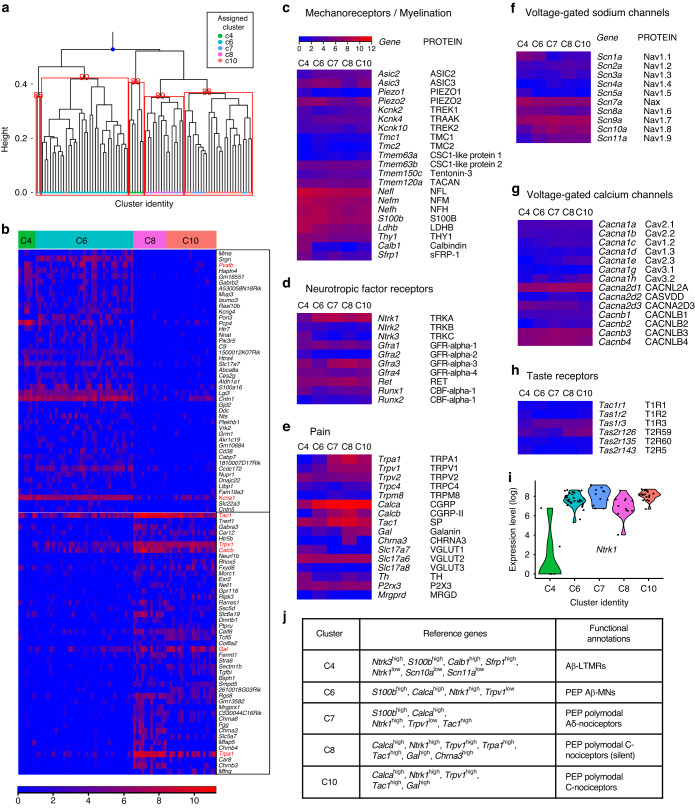
Transcriptome analysis of DPA neurons. **a** Distribution of five major clusters of DPA neurons (excluding C13) through multiscale bootstrap hierarchical clustering analysis. The top node of the hierarchical tree is denoted by as a blue-colored dot. Numbers in red indicate the approximately unbiased (AU) *p*-value for the corresponding cluster. **b** Heatmap displaying the top 45 differentially expressed (DE) genes distinguishing C4/C6 from C8/C10. Gene of interest is highlighted in red (refer to Table S2). **c**–**h** Heatmaps depicting genes in categories relevant to intrinsic properties of somatosensory neurons, including mechanoreceptors/myelination, neurotropic factor receptors, pain, voltage-gated sodium channels, voltage-gated calcium channels, and taste receptors. Data represent the log transform of the mean DESeq2-normalized counts of transcripts in each cluster. Gene names are shown with both gene symbols (italicized, first letter uppercase) and protein symbols (not italicized, all letters uppercase). **i** Transcript levels of *Ntrk1* across DPA clusters. **j** Summary table presenting reference genes and functional annotations of each DPA cluster. LTMRs low-threshold mechanoreceptors, PEP peptidergic, MNs mechanosensitive nociceptors

To assess the robustness of the assigned clusters, we conducted a multiscale bootstrap hierarchical clustering using Euclidean distance on a subset of the 3 000 most variable genes. Bootstrap analysis revealed that the top node of the hierarchical tree distinctly separated C6 from the other clusters, with C4 also forming a separate subtree, distinct from two subtrees containing mixtures of C7/C8 and C7/C10 at a bootstrap replication *p*-value level of 0.05 (Fig. 2a). Thus, while the bootstrap tree did not show significant separation of some of the finer cluster relationships, the separation of C4/C6 from C8/C10 was robustly reproduced. We identified many differentially expressed (DE) genes enriched in C4/C6, such as *Pvalb*, *Kcna1* (Kv1.1), *Scn1a* (Nav1.1), *Scn8a* (Nav1.6), *Ldhb* (Lactate dehydrogenase B), *Nefh* (Neurofilament heavy chain), *S100b*, and *Piezo2*, which are all known for their high expression in myelinated A-fibers^13^ (Fig. 2b and Table S2). Conversely, pain-related DE genes, such as *Trpa1*, *Gal* (Galanin), *Calcb* (Calcitonin gene-related peptide 2), *Trpv1*, *Tac1* (Substance P), *Npy1r* (Neuropeptide Y receptor type 1), *Tlr4* (Toll-like receptor 4), *Calca*, and *Scn11a*, were enriched in C8/C10 (ref. ^28^) (Fig. 2b and Table S2).

To further investigate the subtype classification of DPA neurons, more detailed gene families in somatosensory neurons were examined using the log transform of the mean (DESeq2 normalized) transcript counts in each cluster (Fig. 2c-h). By profiling genes specifically expressed or enriched in each cluster, we assigned clusters of DPA neurons to putative functional subtypes. C4 was assigned to Aβ-fiber LTMRs, marked by high expression of *Calb1* (Calbindin 1, 28 kDa),^11,29^ *Sfrp1* (Secreted frizzled-related protein 1),^27^ and *Ntrk3* (Ref. ^12,27,29^) (Fig. 2c, d), along with low expression of *Scn10a* and *Scn11a*, specifically expressed in nociceptive C-fibers among the voltage-gated sodium channel subtypes^27–29^ (Fig. 2f). This indicates that C4 is not involved in nociceptive functions. Interestingly, with the exception of C4, all other DPA neuron clusters showed high expression of *Ntrk1* (TRKA), which is associated with nerve growth factor (NGF) (Fig. 2d, i). NGF signaling plays an important role in determining the fate of sensory neurons that become nociceptors and increases mechanically activated currents specifically in PEP nociceptive Aδ- and C-fibers.^30,31^ Hence, this result suggests that most DPA neurons possess the potential to function as mechanosensitive nociceptors (MNs) under elevated NGF conditions. Since C6 neurons have high levels of canonical markers for myelinated neurons [e.g., *Nefh*, *S100b*, *Ldhb*, and *Thy1* (Fig. 2c)] and *Calca* (Fig. 2e), but low expression of *Trpv1* (Fig. 2e), we classified them as heat-insensitive Aβ-fiber MNs. This category could further be divided into SA LTMRs and nociceptors, based on their sub-clusters (Fig. 1d-f). C7 neurons similarly expressed myelination markers for myelinated neurons (Fig. 2c), but featured higher expression of nociceptive markers [e.g., *Runx1*, *Trpa1*, *Trpv1*, and *Tac1*] than C6, consistent with the previously established C7 cell type in the TG^8^ (Fig. 2d, e). Consequently, we classified them as intermediate/polymodal Aδ-fiber nociceptors. Finally, C10 and C8 exhibited features associated with polymodal C-fiber nociceptors. Given that C8 and C10 featured high levels of *Gal* and *Tac1* (Fig. 2e), they might represent subclasses of C-fibers transmitting different pain modalities (e.g., nerve injury pain or nociceptive sensitization).^12^ Interestingly, only C8 showed pronounced expression of *Chrna3* (Cholinergic receptor nicotinic alpha 3 subunit), a known marker gene for silent mechanosensitive nociceptors^27,31^ (Fig. 2e), suggesting that this cluster could play a critical role in mechanical nociception under high NGF conditions. With respect to mechanoreceptors, the DPA neurons had low expression of *Piezo1* and *Tmc1* (Transmembrane channel like 1) & *2*, which are associated with itch^32^ and hearing^33^ respectively, but high expression of *Asic3* (Acid-sensing ion channel subunit 3)^34^ and *Piezo2* (ref. ^35^), consistent with previous studies^9,36,37^ (Fig. 2c). Several voltage-gated calcium channels were moderately expressed across all clusters (Fig. 2g). Taste receptors genes were lowly expressed and similarly not specific to any cluster (Fig. 2h). In summary, most DPA subtypes, excluding C4, can be characterized as potent PEP MNs that are affected by NGF signaling (Fig. 2i, j).

### P2X_3_ expression in the PEP subtype of DPA neurons

**Fig. 3:**
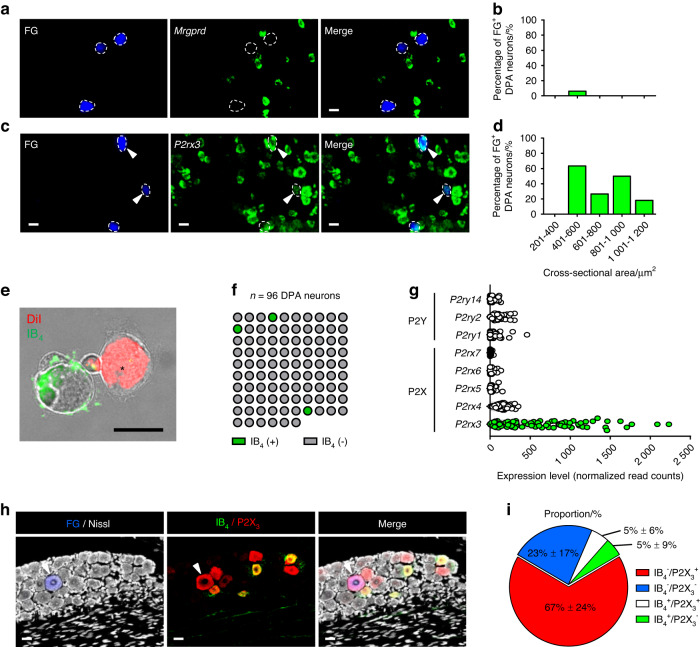
P2X_3_ expression is frequently observed in IB_4_-negative DPA neurons. **a**–**d** Representative fluorescence images (20X magnification) and quantitative results showing DPA neurons labeled with Fluoro-Gold (FG, blue-dotted outline) and each marker gene, *Mrgprd* (C13) and *P2rx3*, shown in green after RNAscope assay. Arrowheads indicate DPA neurons expressing each marker gene. Scale bars: 25 μm. Bar graph represents the cell body size distribution (µm^2^) of DPA neurons expressing *Mrgprd* (*n* = 1 of 62 FG^+^ DPA neurons from *n* = 2 mice) or *P2rx3* (*n* = 12 of 36 FG^+^ DPA neurons from *n* = 2 mice) as the average of two mice. **e** A representative image displaying an IB_4_-negative DiI-labeled DPA neuron (asterisk). A scale bar: 20 μm. **f** The number of IB_4_-positive (*n* = 3 of 96 neurons, green) or IB_4_-negative (*n* = 93 of 96 neurons, gray) DPA neurons during the collection process. **g** Transcript levels of P2Y (G-protein coupled) [e.g., *P2ry1*, *P2ry2*, and *P2ry14*] and P2X (ligand-gated) [e.g., *P2rx3*, *P2rx4*, *P2rx5*, *P2rx6*, and *P2rx7*] purinergic receptor family genes in DPA neuron transcriptomes. **h** Representative immunofluorescence images of FG-labeled DPA neurons (blue) showing P2X_3_ (red) and IB_4_-binding (green) in TG sections. Nissl stain (white) was used for cell body identification. Arrowheads indicate P2X_3_-expressing DPA neurons but do not bind to IB_4_. Scale bars: 25 μm. **i** Proportion of 4 categories [e.g., IB_4_^−^/P2X_3_^+^, IB_4_^−^/P2X_3_^−^, IB_4_^+^/P2X_3_^+^, IB_4_^+^/P2X_3_^−^] among all FG-labeled DPA neurons. Numbers represent mean ± SD. *n* = 100 neurons from *n* = 4 mice

Our FISH assay confirmed that several reference genes from the general TG population were also observed in DPA neurons retrogradely labeled with Fluoro-Gold (FG) (Fig. S3a–h and Fig. 3a–d). In line with nociceptive neurons, *Tac1* and *Trpv1* were predominantly expressed in small to medium-sized DPA neurons (Fig. S3a–d), whereas *Calca* and *Piezo2* displayed high expression across DPA neurons of varying sizes (Fig. S3e–h). Moreover, we consistently observed a low proportion of *Mrgprd*-positive DPA neurons (Fig. 3a, b), in agreement with our sequencing results (Fig. 1b, c and Fig. S1h). *Mrgprd*-positive neurons, which bind to IB_4_, are required for response to noxious mechanical stimuli.^38^ Consistent with previous reports,^9,36^ we observed that the majority of collected DPA neurons did not bind FITC-conjugated IB_4_ (*n* = 3 positive of 96 neurons, Fig. 3e, f), supporting the notion that DPA neurons are primarily composed of PEP nociceptors (Fig. 2j). We also found that a relatively high proportion of DPA neurons exhibited expression of the extracellular ATP ionotropic receptor *P2rx3* (P2X_3_), showing a bimodal distribution across medium to large sizes (Fig. 3c, d). Notably, since *P2rx3* is generally present in NP nociceptors that are distinct from *Trpv1*-positive neurons,^39^ its relatively high expression in DPA neurons, which are *Mrgprd*- and IB_4_-nagative and *Calca*-positive PEP subtypes, is an atypical observation in DRG neurons. Our DPA neuron dataset also showed that *P2rx3* expression predominated over other P2X (ligand-gated) and P2Y (G-protein coupled) purinergic receptors^40^ (Fig. 3g). Our immunohistochemical assays further demonstrated that the majority of P2X_3_-positive DPA neurons did not bind IB_4_-negative (Fig. 3h, i). These findings thus support the notion that ATP, acting through the P2X_3_ receptor, could be a crucial modulator of nociceptive tone in DPA neurons that could contribute to mechanical sensitivity in the context of injury or infection.^41^

### Molecular diversity of MTN neurons innervating the masseter muscle spindles

**Fig. 4:**
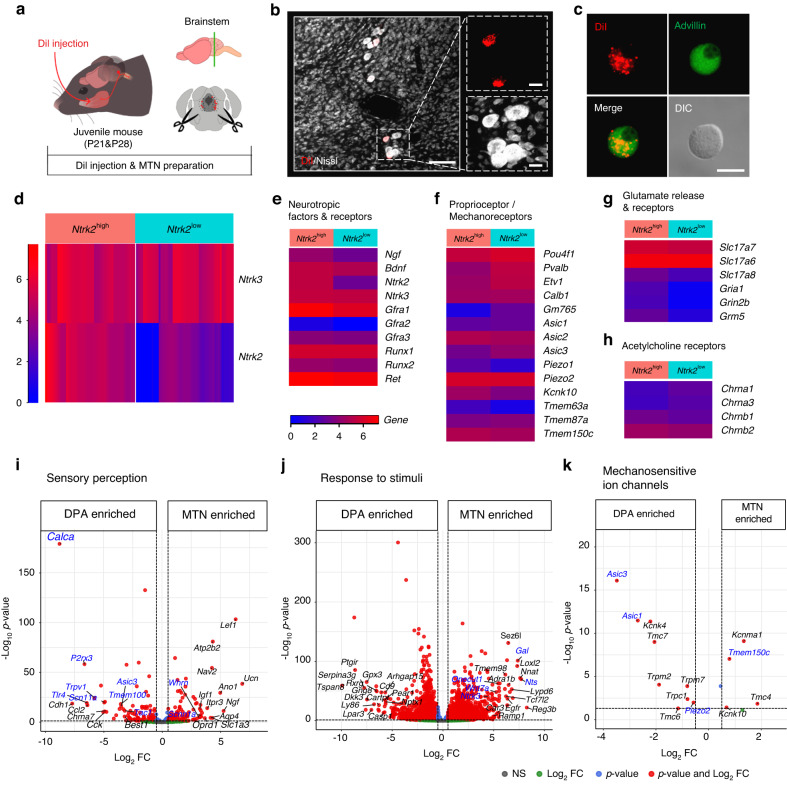
scRNA-seq analysis reveals heterogeneity of mesencephalic trigeminal nucleus (MTN) neurons. **a** Schematic diagram of MTN neuron preparation for scRNA-seq. **b** Representative fluorescence images for a couple of MTN neurons, which were retrogradely labeled with DiI (red) from the masseter muscle, in the brainstem. A scale bar: 100 µm. Insets represent the high-magnification images showing DiI (red) and Nissl staining (white). Scale bars: 20 µm. *n* = 1 mouse (P21). **c** Representative immunofluorescence images showing Advillin positivity (green) in cultured DiI-labeled MTN neurons. A scale bar: 20 µm. *n* = 1 mouse (P21). **d** Heatmap representing the distribution of two major subgroups of MTN neurons: those with high levels of both *Ntrk3* and *Ntrk2* (designated as *Ntrk2*^high^) and those with high levels of *Ntrk3* but low levels of *Ntrk2* (designated as *Ntrk2*^low^). **e**–**h** Heatmaps showing genes categorized according to intrinsic properties, including neurotropic factors and receptors, proprioceptor/mechanoreceptors, glutamate release and receptors, and acetylcholine receptors. Data represent the log transform of mean DESeq2-normalized counts of transcripts in each group (refer to Table S3). **i**–**k** Volcano plots showing the DE genes with a minimum log2 (fold change; FC) of 0.5 and an adjusted *P*-value < 0.05 that are enriched in the DPA neurons (left side) or MTN neurons (right side). For analysis, gene lists from Gene Ontology (GO) categories including sensory perception (GO:0007600), response to stimuli (GO:0050896), and a custom gene list of mechanosensitive ion channels were used. Genes of interest within the top 20 DE genes indicated with text labels), ranked by log2 FC in each GO term, are highlighted in blue (refer to Table S4)

As with DPA neurons, we performed scRNA-seq with the same protocol, but we specifically used juvenile mice to enhance the viability of MTN neurons cultured from brainstem slices (Fig. 4a). For sample preparation, we utilized the retrograde tracer DiI to label cell bodies originating from the masseter muscle spindles in a total of 8 juvenile mice (P21: 5 mice and P28: 3 mice) (Fig. 4a, b) and manually collected DiI-labeled MTN neurons expressing Advillin^20^ (Fig. 4c). Similar to DPA neurons, after quality control, we detected an average of 11 364 genes per MTN neurons from 108 MTN neurons (Fig. S4a–d). The MTN dataset was validated with the expression of specific marker genes associated with proprioceptive neurons [e.g., *Pou4f1* (BRN3A), *Ntrk3*, *Etv1*(ER81), *Pvalb*, and *Whrn* (Whirlin)]^42–44^ in addition to the same set of reference genes that were previously employed for the validation of the DPA dataset^12,13,23,24^ (Fig. S4e). We conducted dimensionality reduction (t-distributed stochastic neighbor embedding; t-SNE) using expression data for the top 3 000 most variable genes, generated by Seurat, in pooled MTN samples of all ages. There was little batch effect between sequencing runs (Fig. S4f).

Proprioceptive neurons innervating muscle spindles are typically classified into two major types: group Ia and group II.^45^ A third type of proprioceptive neuron, group Ib, innervates Golgi tendon organs.^45^ A recent scRNA-seq study focusing on proprioceptive neurons in limb skeletal muscle spindles identified three major marker genes, *Lmcd1*, *Fxyd7*, and *Chad*, representing group Ia, II, and Ib afferents, respectively.^42^ MTN neurons that innervate masticatory muscles are also known to comprise two main afferents, group Ia and II.^7^ Given these similarities, we investigated whether MTN neurons could be similarly classified based on these three marker genes. Our analysis confirmed the presence of comparable subtypes in MTN neurons, namely *Lmcd1*-enriched (similar to group Ia afferents, designated as *Lmcd1*^high^:*Fxyd7*^low^) and *Fxyd7*-enriched (similar to group II afferents, designated as *Lmcd1*^low^:*Fxyd7*^high^) subtypes (Fig. S5a). The cell body size of the *Lmcd1*^low^:*Fxyd7*^high^ subtype tended to be smaller than that of the *Lmcd1*^high^:*Fxyd7*^low^ subgroup, consistent with previous studies^42^ (Fig. S5b). We noted that *Chad* was not detected in our MTN samples, implying that our MTN samples did not contain neurons innervating Golgi tendon.^42^ Additionally, we observed two unidentified subtypes where both markers were enriched or both markers were rarely expressed (Fig. S5a). These four subgroups are visually depicted in a t-SNE plot of MTN neurons (Fig. S5c).

We notably observed the absence of *Runx3* transcripts in MTN neurons (Fig. S4e), despite the predominant expression of other marker genes associated with the early development of MTN neurons [e.g., *Ntrk3*, *Pou4f1*, and *Etv1*].^43,44^ *Runx3* suppresses the expression of *Ntrk2*, a receptor of brain-derived neurotrophic factor (BDNF) that is essential for the development and survival of proprioceptive neurons.^46^ Accordingly, we found that MTN neurons could be categorized into two subgroups with high or low *Ntrk2* expression, termed *Ntrk2*^high^ or *Ntrk2*^low^, respectively (Fig. 4d), and a t-SNE plot visualized the distribution of the two subgroups (Fig. S6a). While the expression levels of neurotrophic factor receptors, with the exception of *Ntrk2* and *Gfra1*, were similar across the two subgroups (Fig. 4d and Table S3), we found higher expression of proprioceptive neuronal marker genes, including *Pvalb* and *Etv1* (ref. ^42^), in *Ntrk2*^low^ subgroup (Fig. 4f and Table S3). This suggests that the *Ntrk2*^low^ subgroup is likely to be representative of typical proprioceptive neurons. Furthermore, we found evidence of glutamate release, which is required for static but not dynamic responses to stretch, and glutamate receptors in MTN neurons^47^ (Fig. 4g and Table S3). We also observed differential expression of nicotinic acetylcholine receptor subunits, which contribute to the sensitivity of a subset of MTN neurons to succinylcholine, an analog of acetylcholine, between the two subgroups (e.g., group Ia: succinylcholine-sensitive or group II: succinylcholine-insensitive)^48^ (Fig. 4h). These results suggest that the *Ntrk2*^high^ subgroup is more likely to exhibit properties associated with glutamate-mediated static activity.^47^ Additionally, we found some differently expressed mechanosensitive ion channels, including *Piezo1* (ref. ^35^) and *Kcnk10* (ref. ^29^) (Fig. 4f and Table S3). Our further analysis revealed many features contributing to their differentiation (Fig. S7a-c).

Next, we conducted a comparative analysis between DPA and MTN neurons to better understand the distinctive characteristics of somatosensation associated with different functions, specifically pain perception and masticatory proprioception. In comparison to MTN neurons, we confirmed that DPA neurons differentially expressed pain-related genes, including *Calca*, *P2rx3, Tlr4*, *Trpv1*, *Scn11a, Asic3*, *Tmem100* (ref. ^49^)*, Tac1*, suggesting that DPA neurons are more involved in nociceptive functions (Fig. 4i and Table S4). Compared to DPA neurons, MTN neurons exhibited higher levels of *Whrn*,^42^ *Scnn1a* (Sodium channel epithelial 1 subunit alpha, regulated by *Etv1*/ER81),^50^ *Onecut1* (One cut homeobox 1, required for normal development of MTN),^51^ *Wnt7a* (Wnt family member 7 A),^12^ *Ntrk3* (ref. ^44^), as well as unexpectedly higher levels of neuropeptide-related genes, such as *Gal* and *Nts* (Neurotensin)^52^ (Fig. 4i, j and Table S4). We also observed differential expression of mechanosensitive ion channels, with higher expression in one or the other cell type (Fig. 4k and Table S4). Overall, these findings support that DPA and MTN neurons possess distinct intrinsic properties for somatosensation.

### MTN neurons experience molecular changes during postnatal development

**Fig. 5:**
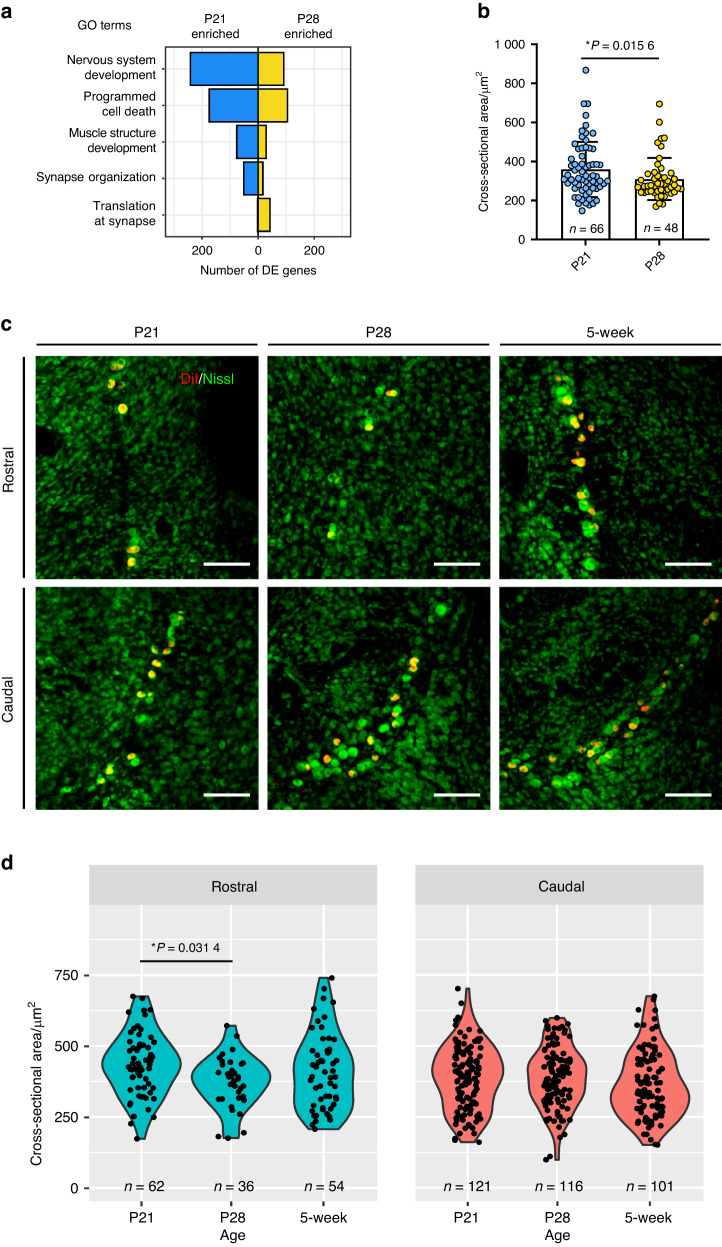
Postnatal changes in MTN neurons. **a** The number of DE genes enriched in P21 (left side) or P28 (right side) of MTN neurons (refer to Table S5). The gene list used for this analysis is within GO categories, including nervous system development (GO: 0007399), programmed cell death (GO: 0012501), muscle structure development (GO: 0061061), synapse organization (GO: 0050808), and translation at synapse (GO: 0140241). **b** Cell body size (μm^2^) of MTN neurons during the collection procedure. P21: *n* = 66 neurons and P28: *n* = 48 neurons. Bar graph presents as the mean ± SD. Unpaired nonparametric test (Kolmogorov-Smirnov test) was performed, *P= 0.015 6. **c** Representative fluorescence images showing DiI-labeled MTN neurons (red) and Nissl stain (green) located in the rostral or caudal part of the brainstem according to mouse age. Scale bars: 100 µm. **d** Violin plots quantitatively depicting neuronal cell body size (μm^2^) by age. *n* = 3 mice (P21), *n* = 2 mice (P28), and *n* = 2 mice (5-week). One-way analysis of variance with Bonferroni post hoc test was conducted, *P= 0.031 4

In rodents, the masseter muscle undergoes considerable changes in morphological properties and activity patterns between the pre-weaning (2 weeks) and post-weaning (4 weeks) stages, which coincide with alterations in feeding behavior.^53,54^ These changes could significantly impact MTN neurons, which coil around the masseter muscle spindles.^55^ This idea was reiterated in a recent study using bulk RNA-seq combined with proteomics in the masseter muscle of mice aged 3 to 25 days, revealing developmental shifts in the molecular composition of muscle spindle capsule cells.^56^ We also sought to investigate the possibility of changes occurring in MTN neurons as a result of the critical behavioral switch from suckling to chewing facilitated by the weaning process in mice. We initially attempted to work with mice younger than 1 week or older than 5 weeks; however, we encountered technical challenges in acquiring labeled neurons from these age groups. After several attempts, we developed an optimized protocol that enables DiI injection into the masticatory muscles of mice at P16 and P23, respectively, followed by cell collection at P21 and P28 (5 days post-injection). Previous research that video-monitored the behavioral patterns of rodent pups from P14 to P35 supports the choice of our P21 and P28 groups, representing an intermediate phase that involves a gradual decrease in suckling behavior followed by an increase in solid food intake within the overall weaning process.^57^ A t-SNE plot displayed the 2-dimensional location of MTN neurons at P21 and P28, respectively (Fig. S6b), and the two subgroups, *Ntrk2*^high^ and *Ntrk2*^low^, showed distinct relative proportions at each postnatal age (Fig. S6c). Moreover, various DE genes were present in biological processes associated with neuron and muscle structure development, such as programmed cell death^58^ and synapse organization^56^ (Fig. 5a). Specifically, DE genes contributing to post-translational processes at synapses were found to be enriched in P28 MTN neurons (Fig. 5a). Furthermore, we observed a significant decrease in the cell body sizes of MTN neurons at P28 compared to P21, both in culture conditions (Fig. 5b) and within a specific subregion of the brainstem, the rostral part (Fig. 5c, d). This may suggest that the postnatal switch is coupled with changes in the cell size of muscle-afferent MTN neurons, which could potentially reflect adaptations to the environment (e.g., external or internal stimuli).

## Discussion

To our knowledge, we are the first to perform scRNA-seq on retrograde-traced DPA and MTN neurons to generate a comprehensive transcriptional overview of oral sensory inputs. This study revealed transcriptional signatures specific to dental sensory transmission of external stimuli, which range from innocuous to noxious, and to proprioceptive transmission of stretching inputs from the masseter muscle spindles. Our single-cell transcriptomic analysis was used to identify cell types and their enriched genes and characterize the landscape of mechanosensory modulation that could be influenced by neurotropic factors such as NGF and BDNF. We could thus determine more specific features of mechanosensory neurons that underlie our perception of pain (nociception) and the regulation of jaw movements (proprioception).

### Why tooth hypersensitivity is evoked by low-threshold mechanical stimulation

In healthy teeth, the tooth pulp innervating nerves are protected by hard tissues, enamel, which covers a layer of dentin around the tooth pulp. However, dental nociception, called *dentin hypersensitivity*, can be initiated in DPA neurons without inflammation through the mechanical stimulation of fluid movement in the dentinal tubules (known as hydrodynamic theory^59^) that have been exposed by tooth fracture or gingival recession. Dentin hypersensitivity produces a sharp, intense pain in response to not only noxious stimuli (e.g., hot, cold, acidic, or hyperosmolar), but also to innocuous mechanical stimuli to the affected tooth, such as air puffs, water spray, and sometimes even a breath.^3,41^ The hydrodynamic theory suggests that DPA neurons differ from other cutaneous sensory neurons where innocuous stimuli do not activate high-threshold nociceptors or evoke pain in a non-injured state.^60^ Several studies to date have demonstrated a specific type of myelinated A-fiber nociceptors (a subset of DPA neurons called *algoneurons*) that secrete CGRP (*Calca*) centrally, which enhances pain transmission.^3,9,36^ Through single-cell transcriptomics analysis, we found that C6/C7 are substantially consistent with algoneuron types, and make up the majority of all DPA neurons we sampled (Figs. 1b, 2j). This result supports the idea that most DPA neurons are myelinated PEP polymodal nociceptors, which can quickly transmit pain signals from all types of stimuli to prevent further damage. Furthermore, since *Calca* was found to be predominantly high throughout DPA neurons compared to other neurotransmitters and peptides involved in pain transmission (Fig. 2e and Fig. S3e, f), CGRP therapeutics for primary headache disorders may be beneficial for cases of intractable dental pain,^61^ although the impact of such therapeutics on concomitant infection and beneficial inflammation will need to be carefully examined.

We also identified a significant presence of the NGF receptor *Ntrk1* that plays a critical role in determining the pathophysiological function of DPA neurons. In general, the expression of *Ntrk1* is essential for cell growth, differentiation, survival, and the fate of sensory neurons to become nociceptors at the embryonic stage, but it has been reported that NGF signaling pathways potentiate electrophysiological properties of low-threshold mechanically activated currents induced by mechanosensitive ion channels in nociceptive PEP Aδ- and C-fibers, but not in NP C-fibers.^30^ In addition, it has been further demonstrated that this property of NGF mediates the acquisition of mechanical sensitivity in “silent” nociceptors (*Chrna3*^+^), normally unresponsive to mechanical stimuli, by upregulating *Piezo2* (ref. ^31^). Thus, C8 with high expression of *Chrna3* could be a strong match for this type (Fig. 2e, j). In addition, given that most DPA neurons have high expression of *Ntrk1* (Fig. 2i, j), with the exception of C4, DPA neurons might have the potential to become sensitized to low-threshold mechanical stimuli by NGF signaling pathways in damaged teeth. NGF is also upregulated in odontoblast-like cells located close to DPA neurons for the repair of teeth and pulp cells in damaged teeth.^62^ This condition gives NGF release from odontoblasts a much greater potential to affect adjacent nerve cells. In addition to their mechanosensory functions, DPA neurons exhibit rapid responses to noxious stimuli, such as heat or cold.^60^ Our analysis demonstrated that the majority of nociceptive DPA neurons are polymodal, given that C7, C8, and C10 shared multiple nociceptors, such as *Trpa1* and *Trpv1* (Fig. 2e). Furthermore, the expression of myelination genes by these nociceptive neurons suggests rapid signaling.

### Intermediate trait of C7

In our bootstrap hierarchical clustering analysis of DPA neurons, C7 exhibited the most diverse distribution, mixed with either C8 or C10 (Fig. 2a). Seurat’s TransferAnchor function assigns a query sample to the cluster from the reference data with the highest prediction score, regardless of the numeric value of the score. Additionally, predictions are limited to known clusters in the reference data; no novel clusters can be identified in the query. When examining the prediction scores per sample, we observed that 30 cells (out of 83) had prediction scores below 0.80. While some of these samples represent cases of possible misassignment between two cluster types, many of the cells demonstrated low prediction scores to multiple clusters, which suggest some DPA neurons may have more ambiguous memberships. Notably, in our data set, every cell predicted to be in C7 had a low prediction score, which led us to hypothesize that C7 represents an intermediary/polymodal cluster (Fig. S2a, b). We note that since these cells were manually isolated, we avoided the possibility of multiplets. While these cells with low and ambiguous prediction scores may simply arise out of noise, they may also represent novel cell types, not in the reference data set or even transition states between cell type clusters. Transition states could arise within our data set either due to variation in the health of labeled mouse pulp (i.e., we cannot rule out prior dental injuries) or as a consequence of neuron dissociation and culture required to recover single neurons.

### Comparison to previously established TG or DPA cell types

We aligned DPA neuron subtypes using the identified cluster information, defining functional subtypes based on published data from the TG as a whole.^8^ To validate our predicted labels, we compared them with the expression patterns of marker genes established in various studies (Fig. 1c, Fig. S1h, and Fig. 2). Emrick and colleagues recently determined that 76% of DPA neurons were C4, 20% were C6, and 4% fell within the other ten clusters defined by whole TG scRNA-seq data,^8^ using FISH assays with eight probes.^9^ Our results are generally in agreement. We found that approximately 50% of DPA neurons fall in C4/C6 while only one was a *Mrgprd*-positive NP C-nociceptors and no *Nppb*- and *Mrgpra3*-positive itch C-nociceptors were identified. However, unlike Emrick and colleagues who determined that C7 neurons are polymodal C-nociceptors,^9^ we believe that DPA neurons in C7 are more likely intermediate/polymodal Aδ-nociceptors based on moderate to high expression of myelinated A-fiber markers [e.g., *Nefh*, *S100b*, *Ldhb*, and *Thy1*] (Fig. 2c) and high expression of nociceptive genes [e.g., *Runx1*, *Trpa1*, *Trpv1*, *Trpm8*, *Calca*, and *Tac1*] (Fig. 2d, e). Therefore, adding to scRNA-seq of TG neurons^8^ and spatial RNA-seq of DRG neurons,^27^ our (increased depth) single-cell transcriptional profiling allowed for a more detailed classification of DPA neurons. However, as we determined the DPA neuron subtypes based on the predicted similarity to the TG cluster types^8^ (Fig. S2a, b) and exclusively examined neurons labeled with the retrograde tracer from molar teeth, we cannot completely exclude the presence of *Mrgprd*-positive DPA neurons based on our inability to find more than one, but they are likely to be rare. Indeed, a previous study has found that *Mrgprd*-green fluorescent protein (GFP) reporter mice exhibited positive signals in the molar pulp, but also only identified 7 cells out of 280 pulp-labeled TG neurons.^63^ Therefore, to confirm these putative classifications, additional experiments following functional assessments such as extracellular single-fiber recordings or in vivo calcium imaging, which can determine fiber-type-specific properties following various stimuli, are required.

### Distinction between two proprioceptive neuronal types innervating the limb muscle spindles and the masseter muscle spindles

Almost all mammalian muscles contain muscle spindles, which are innervated by proprioceptive afferents. These afferents convey sensory information about changes in individual muscle lengths and stretching rates to the CNS, allowing the CNS to perceive the position and movement of our limbs and to regulate motor reflexes.^45^ MTN neurons, which innervate specifically to the masticatory muscle spindles, are comprised of two types of afferents, named group Ia and group II, according to their axonal conduction velocity.^7^ Group Ia afferents can be distinguished from group II afferents by their lower activation threshold, faster conduction velocity, and dynamic sensitivity.^7,48^ However, since MTN neurons exhibit a wide range of conduction velocities, it is challenging to practically differentiate between the two types of afferents.^48^ Another categorization of MTN neurons based on acetylcholine sensitivity has been proposed in previous studies, but this specific phenotype was observed in cats rather than rats.^48,64^ Therefore, to identify the molecular classification of MTN neurons, we first focused on the three major marker genes, such as *Lmcd1*, *Fxyd7*, and *Chad*,^42^ corresponding to group Ia, II, and Ib afferents, respectively, previously identified in limb skeletal muscle spindles. Our results showed that these genes could be applied to a subset of MTN neurons to help identify their subtypes (Fig. S5a). In addition, typical proprioceptive neuronal markers such as *Pou4f1* and *Ntrk3*, were predominantly expressed in MTN neurons, whereas the transcript levels of other marker genes *Etv1*, *Pvalb*, and *Whrn* were highly heterogenous or rarely expressed (Fig. S4e). More surprisingly, *Runx3*, a representative marker for proprioceptive neurons, was also not found in MTN neurons (Fig. S4e). These findings are worth noting, given that proprioceptors have a mechanism to maintain proprioceptors by *Runx3* suppression of *Ntrk2* expression.^46^ However, the *Ntrk2*-negative traits are another important marker for classifying proprioceptive neurons in DRG neurons; therefore, careful consideration should be given to whether our results might be a temporary phenomenon that appears at a certain age of mice. These findings may also be an effect of their CNS localization, as *Ntrk2* is the BDNF receptor. We were able to divide MTN neurons into two subgroups based on expression levels of *Ntrk2* (Fig. 4d and Fig. S6a). We also found that *Ret*, a representative marker of Aβ SA LTMRs,^29^ was highly expressed throughout MTN neurons (Fig. 4e), suggesting that MTN neurons possess molecular features of both proprioceptors and Aβ SA LTMRs in response to stretch stimuli. It remains to be determined whether and how the *Ntrk2*^high^ and *Ntrk2*^low^ subgroups are related to group II and group Ia afferent, respectively. It should be noted that *Ntrk2* levels are on a continuum, so subtle differences in its expression can affect classification. Furthermore, there is a possibility of differential expression of unidentified mechanosensitive molecules or mechanosensitive ion channels (namely transmembrane (TMEM) protein family), such as *Tmem150c* (Tentonin-3),^65^ between the two subgroups. Further research is needed to elucidate and understand these potential factors. Taken together, our deeply sequenced datasets not only increase our understanding of proprioceptive MTN neurons but also provide an important resource for exploring additional candidate genes responsible for aspects of mechanosensation.

### Limitations of this study

This study takes into consideration the influence of in vitro experimental conditions, which could induce damage or stress-derived transcriptional factors and persistent morphological changes by neurite growth. It is also possible that the absence of neurotrophic factors that are crucial for the maintenance and survival of somatosensory neurons in vivo may affect our results. In addition, the presence of gene expression generally used for glial markers,^23^ such as *Gfap*, *Kcnj10*, and *Gja1*, in certain DPA and MTN neurons raises the possibility of glial cell contamination during the cell collection procedure. However, these samples included for the downstream analysis as glial marker gene expression did not significantly influence the classification of neuron subtypes. Interestingly, these markers were found to be more frequently expressed in P21 MTN neurons compared to DPA neurons or P28 MTN neurons. Recently, snRNA-seq and spatial transcriptomics have emerged as a powerful tool for characterizing somatosensory neurons under various conditions, including injury or pain,^15,27^ so further studies with this technology will be valuable to understand these neuronal population.

In conclusion, our transcriptome analysis revealed a new classification in which DPA neurons have a unique capacity to transmit mechano-nociceptive signals in response to innocuous mechanical stimuli in teeth, and MTN neurons exhibit distinct responses to mechanical stretching in the masseter muscle. These characteristics were based on the expression patterns of the neurotrophic factor receptors *Ntrk1*/TRKA, *Ntrk2*/TRKB, and *Ntrk3*/TRKC. The precise regulation of these receptors plays a critical role in conferring unique properties to the pain- and mechano-transducers of the dental and masseter nerves, setting it apart from the bodily somatosensory system.

## Materials and methods

### Animals

A total of 30 adults (5- to 7-week old) and 15 juvenile (P16 to P28) male C57BL/6J mice were obtained from the Doo Yeol Biotech (Republic of Korea) and Jackson Laboratories (Bar Harbor, Maine, USA). All surgical and experimental procedures were approved by the Institutional Animal Care and Use Committee (IACUC) at Seoul National University and University of Pennsylvania and conformed to the ARRIVE (Animal Research: Reporting In Vivo Experiments) guidelines for animal studies.

### Retrograde labeling of neurons and single-cell isolation

DPA neurons were retrogradely labeled with a few 1,1′-Dioctadecyl-3,3,3′,3′-tetramethylindocarbocyanine perchlorate (DiI, Invitrogen, USA) crystals from the maxillary first molars on both sides in each mouse (5-week-old), then prepared as described previously.^66^ Two weeks post-labeling, DPA neurons were isolated in acute culture from the TG tissues as previously described.^66^ DPA neurons were incubated with FITC-conjugated isolectin B4 (10 μg·mL^−1^ in culture media, Sigma, USA) for 15 min at 37 °C to detect NP C-fibers. Similarly, MTN neurons were retrogradely labeled with a total volume of 5 to 10 µL DiI (3% in Dimethyl sulfoxide; DMSO) from the masseter muscles on both sides in each mouse (either P16 or P23). Five days post-labeling, mice were sacrificed, and the brainstem tissue from the midbrain to the medulla oblongata was dissected out and embedded in agarose. The tissue was cut into 300 to 500 μm thickness coronal slices with a vibrating blade microtome (VT1000P, Leica, USA) while immersed in an artificial cerebrospinal fluid solution. Unlike DPA neurons, MTN neurons should be dissociated from brainstem slices, so we used young mice in order to increase cell viability.^67^ MTN neurons were acutely cultured by modifying a previous method used for rats^67^ to be more appropriate for mice. Briefly, we placed chunks of MTN tissue in papain solution (20 U·mL^−1^, Worthington Biochemical, USA) in Ca^2+^- and Mg^2+^-free Hanks’ Balanced Salt Solution (HBSS, Gibco, USA), added a few crystals of L-cysteine (Sigma, Germany), adjusted to pH 7.4, and incubated the tissue chunks for 15 to 25 min at 37 °C. After aspirating fluid, we added Ham’s F12 culture media (Gibco, USA) containing 10% fetal bovine serum (FBS), 1% Penicillin/Streptomycin (P/S), and NGF 2.5S (50 ng·mL^−1^, Invitrogen, USA) and triturated the tissues extremely gently until chunks were diminished. Afterward, cells were placed on poly-D-lysine/laminin-coated glass coverslips, and culture media were changed to Ham’s F12 feeding media containing 10% FBS and 1% P/S but no neurotrophic factors. Cells were incubated for at least four hours at 37 °C (5% CO_2_) in a humid incubator until the start of the cell collection.

### scRNA-seq

#### Sample collection

To minimize cell-to-cell variation under in vitro conditions, cells were collected only during the 6-hour incubation period from the start of cell collection. During the collection procedure, we continuously flowed a freshly prepared diethyl pyrocarbonate (DEPC, Sigma, Switzerland)-treated 2 mM Ca^2+^/Na^+^ solution (bath solution). DPA and MTN neurons were manually collected using a glass micropipette filled with a pipette solution containing RNase inhibitor (2 U·μL^−1^, Takara, China) in bath solution while minimizing non-neuronal contaminations. Each neuron was photographed prior to collection, and the images were used for cell body area (μm^2^) measurements using ImageJ software (National Institutes of Health, USA). We also collected the bath solution without cells as the negative control for each batch.

#### cDNA library preparation and sequencing

Once the DPA neurons were separated into individual tubes, they were digested and reverse-transcribed to non-stranded cDNA using poly-T primers. The resulting cDNA was amplified using SMART-Seq v4 Ultra Low Input RNA kit (Clontech, China) into cDNA libraries according to the manufacturer’s instructions, but with the following modifications: 1) ERCC RNA Spike-In controls (1/4 000 000 dilution, Invitrogen, Lithuania) were added at the cell lysis step; 2) 18 PCR cycles were performed to amplify cDNA libraries. The libraries were diluted accordingly and used to make DNA sequencing libraries by following the Nextera XT DNA Library Preparation Kit protocol (Illumina, USA). Before sequencing, the libraries were analyzed using a Bioanalyzer High Sensitivity DNA Kit (Agilent Technologies, USA) to determine their quality. Libraries that passed quality control filters (for size distributions) were sequenced using a NextSeq 500 (Illumina, USA) with single-end 75 base pair reads. Reads were trimmed for adapters and poly-A sequences using in-house software and then mapped to the mouse genome (mm10) using STAR.^68^ In the resulting dataset, the average sample has a depth of 5.8 million reads, with 4.1 million uniquely aligned to exons. Uniquely mapped reads were used for feature quantification using VERSE.^69^

#### Sample quality control

We collected a total of 96 DPA neurons from 10 mice (7-week-old) in two independent batches. A total of 75 MTN neurons (P21, 5 mice) and 47 MTN neurons (P28, 3 mice) were collected in single batches separated by age. Based on summary statistics from in-house software, 10 samples with “Percent Spike-In” > 5%, indicating relatively low RNA concentration compared to the spike-in standard, were removed from downstream analyses. Also, three additional samples with a concentration of less than 0.5 ng·μL^−1^ after reverse transcription and amplification were removed. Consequently, a total of 83 DPA samples were used in the downstream analysis. Similarly, from the MTN neurons, two samples that had “Percent Spike-In” > 5%, two samples that had “Percent mitochondrial transcript” > 10%, and two samples that had a concentration of less than 0.5 ng·μL^−1^ after reverse transcription and amplification were removed. Also removed were four additional samples without expression of *Avil* and another four samples with no detectable expression of *Ntrk3*, *Etv1*, or *Pvalb* all previously known markers of proprioceptive neurons.^42^ Consequently, a total of 108 MTN samples were used in the downstream analyses.

### scRNA-seq data analysis

#### DPA cluster identification

This paper made extensive use of the Seurat package developed by the Satija lab^70^; in essence, the recommended (TransferData) methods were used to project reference data onto a query object.^25^ Briefly, single-cell TG transcriptome data, to be used as the reference,^8^ were log-normalized and center scaled; variable features were identified and used for linear dimensional reduction (principal components analysis; PCA). As previously described, informative principal components were used for clustering, and multidimensional data were displayed in a t-SNE 2-dimensional representation. Similarly, DPA transcriptome data (query) were log-normalized and center scaled; variable features were identified. Next, anchors were identified in the reference data set, and a weights matrix was constructed that defined the association between each query cell and each anchor. Afterward, a binary classification matrix was created, where the rows correspond to possible cluster identities, and the columns correspond to the anchors, and each matrix cell is 1 if the anchor pair is a member of a certain cluster. The classification matrix was then multiplied by the weights matrix as mentioned above to calculate prediction scores to transfer cluster labels onto the DPA data. Each query sample was assigned to the cluster with the highest prediction score. When transferred cluster labels were visualized in a 2-dimensional non-linear dimensionality reduction (t-SNE) of the top 3 000 most variably expressed genes (normalized by DESeq2), then log transformed. The neurons were well separated into their respective assigned clusters.

We wanted to verify the validity of the transferred labels with an independent clustering method, using the same subset of log-transformed, DESeq2-normalized counts of the top 3 000 variable features. Given the limited number of samples compared to that of droplet-based capture and sequencing and the uncertainty of hierarchical clustering, we used R package pvclust^71^ to perform multiscale bootstrap hierarchical clustering. Briefly, pvclust generates bootstrap samples by randomly sampling varying proportions of the data, then computes a hierarchical clustering on each bootstrap copy. Then, for each cluster observed in the hierarchical clustering of the original data, it calculates a *P*-value between 0 and 1, corresponding to how strongly the data supports the cluster, based on how often the cluster was observed across hierarchical clustering of the bootstrap copies. Based on the approximately unbiased (AU) *p*-values in the bootstrap hierarchical clustering, there we found strong evidence of clusters composed of samples assigned to a single cluster, except for samples predicted to be C7, as well as separation of samples across C4/C6 and C8/C10, with a single exception. To summarize, this multiscale bootstrap hierarchical clustering analysis independently reproduces the transferred cluster label from Seurat.

#### Differentially expressed (DE) gene analysis

For differential expression analysis, only genes that contained at least three reads across five samples were considered. Read counts were normalized for library size using DESeq2 and DE genes were identified with DESeq2. A minimum fold change of 1.2 and an adjusted *P*-value < 0.05 after multiple hypothesis correction (Benjamini–Hochberg) was used to identify significant DE genes. Gene set enrichment analysis (GSEA) was performed using the R package clusterProfiler (gseGO) ^72^.

### Fluorescence in situ hybridization (FISH)

#### Retrograde labeling of DPA neurons

Since the lipophilic dye DiI was inadequate for the FISH method, we used another retrograde neuronal tracer Fluoro-Gold (FG, Cayman Chemical, USA) for labeling DPA neurons. All procedures of tooth dentin and pulp exposure surgery, including anesthesia, anti-inflammatory analgesic treatment, and surgery in mice, were followed as previously described.^73^ A total volume of 0.5–2 µL FG (4% in distilled water) was directly applied to an exposed dental pulp of the maxillary molar (either a left side or both sides of the jaw) in mice, and then the dental cavity was sealed with the GC Fuji II dental cement (Cat. #21, GC Corporation, Japan) according to the manufacturer’s instructions. Upon anesthetic recovery, animals were returned to their home cages.

#### Sample preparation and RNAscope assay

Five days post-labeling, mice were deeply anesthetized and were perfused with phosphate-buffered saline (PBS) solution, followed by ice-cold 4% paraformaldehyde in PBS (PFA solution). TG tissues were post-fixed in 4% PFA solution for 4 h at 4 °C and then were transferred to 30% sucrose solution in PBS at 4 ℃. Serial frozen transverse sections (thickness: 14 µm) of TG were mounted onto superfrost plus slides and were stored at −80 °C until later use. FG-positive cells were verified microscopically prior to FISH. The sections were used for RNAscope Multiplex Fluorescent v2 assay (Advanced Cell Diagnostics, USA), as previously described,^73^ using the following probes: Mm-*Tac1* (Cat. #410351-C2), Mm-*Trpv1* (Cat. #313331-C2), Mm-*Calca* (Cat. #420361-C2), Mm-*Piezo2* (Cat. #400191-C3), Mm-*Mrgprd* (Cat. #417921-C2), and Mm-*P2rx3* (Cat. #521611-C3). All z-stack images were acquired using a fluorescence microscope (Leica) controlled by LasX software.

#### RNAscope assay image analysis

Cell body area (µm^2^) of labeled neurons was all measured with ImageJ. In particular, since the FG signal of DPA neurons was significantly weakened by wash steps in the RNAscope assay, we took images of FG-positive cells using the DAPI filter cube before the assay, then imaged again after the assay with different probe images using the Cy3 or Cy5 filter cube, using memory function in the microscope to ensure the same field of view was captured both times. We used Fiji Plugins of ImageJ to align and merge FG images (in the DAPI range) with the corresponding probe image (in the Cy3 or Cy5 range) obtained after the assay. A total of three to six DPA sections for each probe were randomly selected and converted to 8-bit grayscale images, and the brightness/contrast and threshold adjustments were applied to each fluorophore channel separately. Positive neurons were determined if there were at least 10 puncta dots inside the FG-positive somata or if their average intensity values were above the threshold.

### Immunocytochemistry

Cells on poly-D-lysine-coated glass coverslips were washed three times with ice-cold PBS, followed by fixation with 4% PFA solution for 15 min. After washing three times with PBS for 5 min each, the cells were incubated with a blocking solution containing 2% bovine serum albumin (BSA) and 0.2% Tween 20 at room temperature (RT) for 1 h, and then anti-advillin antibodies (1:1 000, Cat. #ab72210, Abcam, USA) diluted in blocking solution were incubated overnight at 4 °C. The next day, cells were washed with PBS containing 0.1% Tween 20 for 10 min, washed twice with PBS for 5 min each, then were co-incubated with donkey anti-rabbit Alexa Fluor 488 (1:200, Cat. #A-21206, Invitrogen, USA) and DAPI (1:1 000, Cat. #D9542, Sigma, Israel) diluted in PBS at RT for 2 h. After washing in the same way, the coverslips were mounted with a mounting medium (Vector Laboratories, USA). All images were examined using a confocal microscope (LSM700, Carl Zeiss Microscopy, Germany).

### Immunohistochemistry

Mice were deeply anesthetized and were perfused with PBS, followed by ice-cold 4% PFA solution. TG and brainstem tissues were dissected out, post-fixed in 4% PFA solution at 4 °C overnight, and then transferred to 30% sucrose solution in PBS at 4 °C. We prepared TG and brainstem sections, which were transversely sectioned (TG thickness: 14 µm and brainstem thickness: 30 to 40 µm). For experiments in Fig. S1a, Fig. 4b, and Fig. 5c, the sections were washed three times with PBS for 10 min each. For permeabilization, the sections were washed with PBS containing 0.1% Tween 20 for 10 min, washed twice with PBS for 5 min each, and then incubated with NeuroTrace 500/525 Green Fluorescent Nissl Stain (1:100, Invitrogen, USA) diluted in PBS for 30 min at RT. After washing in the same way, the slides were mounted with the mounting medium. All images were examined using a confocal microscope. For subsequent immunohistochemical analyses in Fig. 5c, d, a total of two to three sections corresponding to the rostral part (Bregma −4.9 mm to −5.1 mm) and caudal part (Bregma −5.4 mm to −5.5 mm) of the brainstem of each mouse were randomly selected. The images were converted to 8-bit grayscale and analyzed with ImageJ for their cross-sectional area (µm^2^) measurements.

For experiments in Fig. 3h, i, TG sections were washed three times with PBS for 10 min each and were incubated with a blocking solution containing 10% normal donkey serum (NDS) and 0.3% Triton X-100 in PBS for 1 h at RT and followed by rabbit anti-P2X_3_ receptor (1:1 000, Cat. #APR-016, Alomone labs, Israel) and Isolectin B_4_, biotin conjugate (20 μg·mL^−1^, Cat. #L2140, Sigma, Israel) in PBS containing 1% NDS and 0.3% Triton X-100 for overnight at 4 °C. On the next day, the slides were washed with PBS containing 0.1% Triton X-100 for 10 min and washed twice with PBS for 5 min each, followed by donkey anti-rabbit Alexa Fluor 594 (1:200, Invitrogen, USA), Alexa Fluor 488 Streptavidin (1:500, Jackson ImmunoResearch, USA), and NeuroTrace 640/660 Deep-Red Fluorescent Nissl Stain (1:100, Invitrogen, USA) incubated in PBS for 2 h at RT. After washing in the same way, the slides were mounted with the mounting medium. Images were obtained using a confocal microscope, and all images were converted to 8-bit grayscale with ImageJ, brightness/contrast and color thresholds were equalized, and positive neurons were counted in three to six sections obtained from each mouse.

### Statistics and data presentation

Bar graphs/statistical data are presented as the mean or mean ± SD. Comparisons between the two groups in Fig. 5b were analyzed using an unpaired nonparametric test, and comparisons between the three groups in Fig. 5d were analyzed using a one-way analysis of variance. Differences were considered statistically significant at *P*-value < 0.05. Bar graphs and plots were generated using the Prism software (v.7.00, GraphPad), RStudio (v.1, RStudio, Inc.), or R (v.4, R Project for Statistical Computing).

### Supplementary information

Supplementary table titles and Supplementary figure legends

Table S1, Table S2, Table S3, Table S4 & Table S5

Fig. S1, Fig. S2, Fig. S3, Fig. S4, Fig. S5, Fig. S6 & Fig. S7

## Data Availability

The raw read count data of genes, which are provided in the supplementary tables, are supplied in the supplementary information. The datasets supporting the conclusions of this article are available in the GEO and SRA repositories under accessions (GSE216835).
