# Supplementary material for: Transcriptional profiling of dental sensory and proprioceptive trigeminal neurons using single-cell RNA sequencing
Source: Int J Oral Sci. 2023 Sep 25;15:45. doi: 10.1038/s41368-023-00246-z (PMC10519964; doi:10.1038/s41368-023-00246-z)
Supplement: Supplementary file 1 — Supplementary table titles and Supplementary figure legends [file 41368_2023_246_MOESM1_ESM.docx]

**Supplementary Information**

**Transcriptional profiling of dental sensory and proprioceptive trigeminal neurons using single-cell RNA sequencing**

**Running title: scRNA-seq of dental and masseter nerves**

Pa Reum Lee, Jihoon Kim, Heather Lynn Rossi, Sena Chung, Seung Yub Han, Junhyong Kim, Seog Bae Oh

**Supplementary table titles**

Supplementary Table 1. Differentially expressed (DE) genes between C6A and C6B DPA neurons, related to Fig. 1e-f.

Supplementary Table 2. DE genes between C4/C6 and C8/C10 DPA neurons, related to Fig. 2b.

Supplementary Table 3. DE genes between two categories of MTN neurons: *Ntrk2*^high^ and *Ntrk2*^low^, related to Fig. 4d and Fig. S7.

Supplementary Table 4. DE genes between DPA and MTN neurons, related to Fig. 4i-k.

Supplementary Table 5. DE genes between P21 and P28 of MTN neurons, related to Fig. 5a.

**Supplementary figure legends**

Supplementary Fig. 1. Assessment of transcriptomes in DPA neurons. **a** Representative fluorescence images showing a couple of DPA neurons, which were retrogradely labeled with DiI (red) from a maxillary first molar, in the maxillary branch of the trigeminal ganglion (TG). A scale bar: 100 µm. Insets represent the high-magnification images showing DiI (red) and Nissl staining (white). Nissl stain was used for cell body identification. Scale bars: 20 µm. *n* = 1 mouse. **b** Representative immunofluorescence images showing Advillin positivity (green) in cultured DPA neurons labeled with DiI (red). A scale bar: 20 µm. *n* = 1 mouse. **c-f** A total of 83 DPA neurons’ transcriptomes having a depth of 5 ± 1.1 million (mean ± SD) with 3.8 million uniquely aligned exonic reads (c), data-detected genes (11,915 ± 2697, d), percentage of Spike-In (e), and percentage of mitochondrial transcript (f) per neurons. **g** Heatmap showing transcript expression levels of reference genes across 83 DPA neurons, including housekeeping genes [e.g., *Gapdh* and *Actb*], sensory neuronal markers [e.g., *Tubb3*, *Uchl1*, *Avil*, and *Rbfox3*], glial markers [e.g., *Gfap*, *Kcnj10*, and *Gja1*], and motor neuronal markers [e.g., *Chat* and *Neurog2*]. Raw counts (not DESeq2 normalized) have been log transformed. Of note, most DPA samples did not express glial markers or motor neuronal markers. **h** t-SNE plots expressing key marker genes detailed in the previous studies on TG samples^1,2^ in assigned DPA neuron clusters. **i** Cell body size (μm2) of identified DPA neuronal types as measured on cultured cells during the collection procedure.

Supplementary Fig. 2. Intermediate nature of C7 neurons. **a** Graph showing prediction scores to cluster labels onto the DPA datasets. **b** A t-SNE plot of DPA neurons belonging to C7 low prediction scores (low prediction scores; LPS) as represented by grey dots.

Supplementary Fig. 3. Validation of selected marker genes in DPA neurons using the RNAscope assay. **a-h** Representative fluorescence images (20X magnification) of DPA neurons labeled with Fluoro-Gold (FG, blue-dotted outline) and each marker gene, such as *Tac1* (C7/C8/C10), *Trpv1* (C7/C8/C10), *Calca* (C6/C7/C8/C10), and *Piezo2* (C4/C6), shown in green after RNAscope assay, are presented in panels a, c, e, and g. Arrowheads indicate DPA neurons expressing each marker gene. Scale bars: 25 μm. Cell body size distribution (µm^2^) of DPA neurons expressing *Tac1* (*n* = 7 of 42 FG^+^ DPA neurons from *n* = 2 mice), *Trpv1* (*n* = 17 of 81 FG^+^ DPA neurons from *n* = 2 mice), *Calca* (*n* = 22 of 58 FG^+^ DPA neurons from *n* = 2 mice), and *Piezo2* (*n* = 59 of 81 FG^+^ DPA neurons from *n* = 2 mice) are shown in panels b, d, f, and h. Bars represent averages from two mice per marker.

Supplementary Fig. 4. Quality control of MTN neurons. **a-d** A total of 108 MTN neurons’ transcriptomes having a depth of 6.3 ± 1.8 million (mean ± SD) with 4.5 million uniquely aligned axonic reads (a), data-detected genes (11,324 ± 2116, b), percentage of Spike-In (c), and percentage of mitochondrial transcript (d) per neurons. **e** Heatmap showing transcript expression levels of reference genes across 108 MTN neurons (P21: 67 neurons and P28: 41 neurons), including housekeeping genes [e.g., *Gapdh* and *Actb*], sensory neuronal markers [e.g., *Tubb3*, *Uchl1*, and *Avil*], proprioceptive neuronal markers [e.g., *Pou4f1*, *Ntrk3*, *Etv1*, *Pvalb*, and *Whrn*], glial markers [e.g., *Gfap*, *Kcnj10*, and *Gja1*], and motor neuronal markers [e.g., *Chat* and *Neurog2*]. Of note, among proprioceptive neuronal marker genes, the transcript levels of *Etv1*, *Pvalb*, *Whrn, and Runx3* displayed considerable heterogeneity across MTN neurons. While most samples did not express motor neuronal marker genes, the expression of glial markers showed distinct patterns, particularly between P21 and P28 age groups. **f** A t-SNE plot showing no batch effect between sequencing runs.

Supplementary Fig. 5. Expression of two major proprioceptive marker genes identified in limb skeletal muscle spindle afferents. **a** Heatmap showing the expression of *Lcmd1* and *Fxyd7*, which are markers representing group Ia and II afferents, respectively, identified in limb skeletal muscle spindles^3^, in MTN samples. At least four subgroups were observed: *Lcmd1*^high^:*Fxyd7*^low^, *Lcmd1*^low^:*Fxyd7*^high^, *Lcmd1*^high^:*Fxyd7*^high^, and *Lcmd1*^low^:*Fxyd7*^low^. **b** Cell body size (μm^2^) of MTN neurons as measured on cultured cells during the collection procedure. **c** A t-SNE plot showing these four subgroups of MTN neurons.

Supplementary Fig. 6. t-SNE plots of MTN neurons. **a** A t-SNE plot showing the distribution of the two MTN neuron subgroups, *Ntrk2*^high^ and *Ntrk2*^low^. **b** A t-SNE representation of MTN neurons depicting using two colors to differentiate between the two ages. **c** The percentages of MTN neurons from each subgroup on each age are provided: P21, *Ntrk2*^high^: 64% (*n* = 43 of 67 neurons), P21, *Ntrk2*^low^: 36% (*n* = 24 of 67 neurons), P28, *Ntrk2*^high^: 22% (*n* = 9 of 41 neurons), and P28, *Ntrk2*^low^: 78% (*n* = 32 of 41 neurons).

Supplementary Fig. 7. Comparison of *Ntrk2*^high^ and *Ntrk2*^low^ subgroups of MTN neurons. **a-c** Dot plots showing the gene set enrichment analysis (GSEA) results for *Ntrk2*^high^ *vs.* *Ntrk2*^low^ subgroups according to biological process (BP in panel a), cellular compartment (CC in panel b), and molecular function (MF in panel c). Significantly enriched GO terms were determined based on a nominal *p*-value and false discovery rate (FDR) < 5% as the cutoff. The circle size represents the proportion of cells expressing the marker gene in a cluster, and the color intensity reflects its average expression level within that cluster.

**References**

1 Nguyen, M. Q., Wu, Y., Bonilla, L. S., von Buchholtz, L. J. & Ryba, N. J. P. Diversity amongst trigeminal neurons revealed by high throughput single cell sequencing. *PLoS One* **12**, e0185543 (2017). https://doi.org:10.1371/journal.pone.0185543

2 von Buchholtz, L. J., Lam, R. M., Emrick, J. J., Chesler, A. T. & Ryba, N. J. P. Assigning transcriptomic class in the trigeminal ganglion using multiplex in situ hybridization and machine learning. *Pain* (2020). https://doi.org:10.1097/j.pain.0000000000001911

3 Wu, H. *et al.* Distinct subtypes of proprioceptive dorsal root ganglion neurons regulate adaptive proprioception in mice. *Nat Commun* **12**, 1026 (2021). https://doi.org:10.1038/s41467-021-21173-9
