# Supplementary material for: Transcriptional profiling of dental sensory and proprioceptive trigeminal neurons using single-cell RNA sequencing
Source: Int J Oral Sci. 2023 Sep 25;15:45. doi: 10.1038/s41368-023-00246-z (PMC10519964; doi:10.1038/s41368-023-00246-z)
Supplement: Supplementary file 3 — Fig. S1, Fig. S2, Fig. S3, Fig. S4, Fig. S5, Fig. S6 & Fig. S7 [file 41368_2023_246_MOESM3_ESM.pptx]

## Slide 1
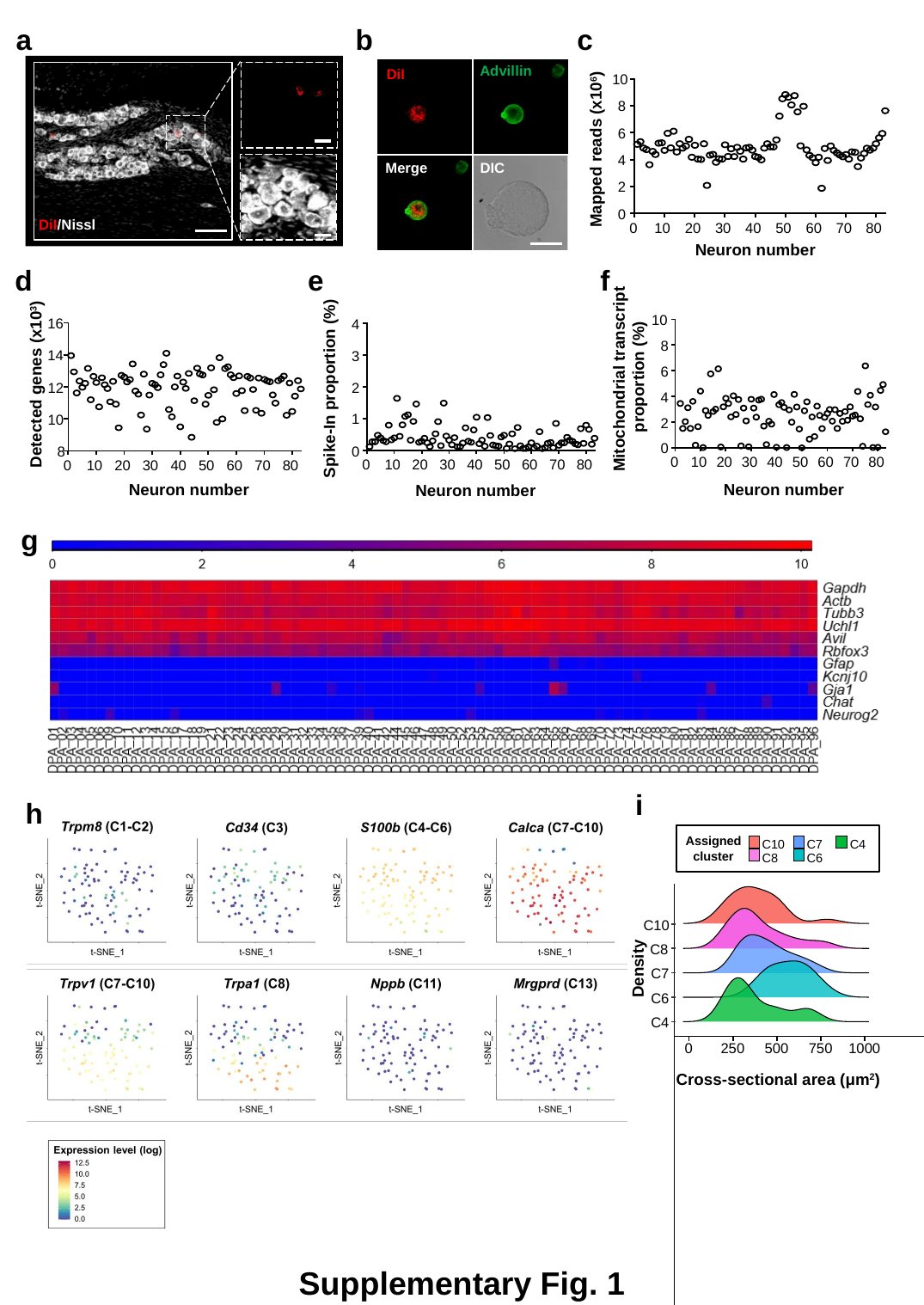

a
b
c
DiI/Nissl
Advillin
DiI
DIC
Merge
10
8
6
Mapped reads (x106)
4
2
0
0
10
20
30
40
50
60
70
80
Neuron number
d
e
f
10
8
Mitochondrial transcript
 proportion (%)
6
4
2
0
0
10
20
30
40
50
60
70
80
Neuron number
4
3
2
Spike-In proportion (%)
1
0
0
10
20
30
40
50
60
70
80
Neuron number
16
14
Detected genes (x103)
12
10
8
0
10
20
30
40
50
60
70
80
Neuron number
g
i
h
C10
C7
C4
Assigned
cluster
C8
C6
C10
C8
C7
Density
C6
C4
250
500
750
0
1000
Cross-sectional area (μm2)
Supplementary Fig. 1

## Slide 2
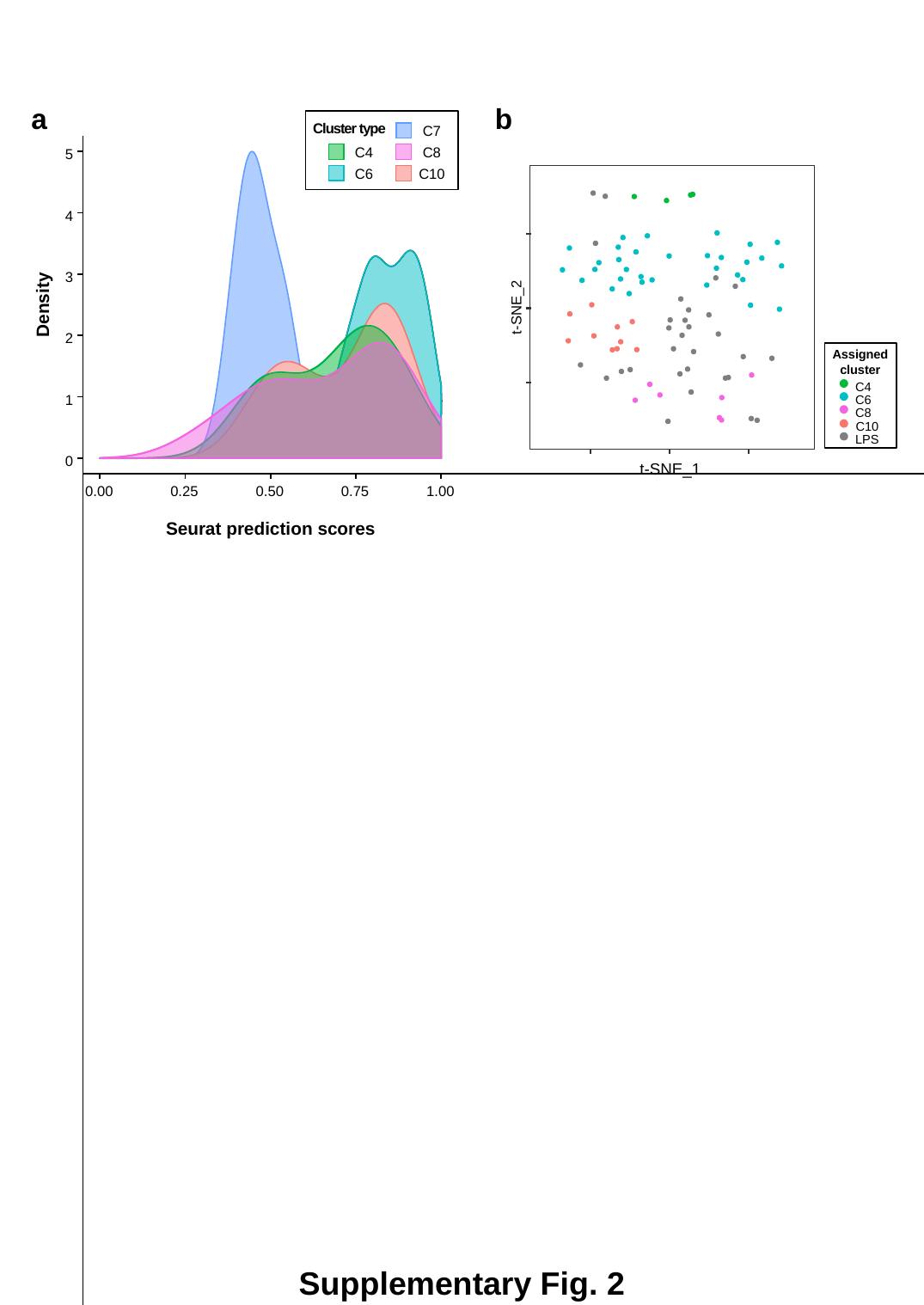

a
b
Cluster type
C7
C4
C8
C6
C10
5
4
3
Density
2
1
0
0.25
0.00
0.50
0.75
1.00
Seurat prediction scores
t-SNE_2
Assigned
cluster
C4
C6
C8
C10
LPS
t-SNE_1
Supplementary Fig. 2

## Slide 3
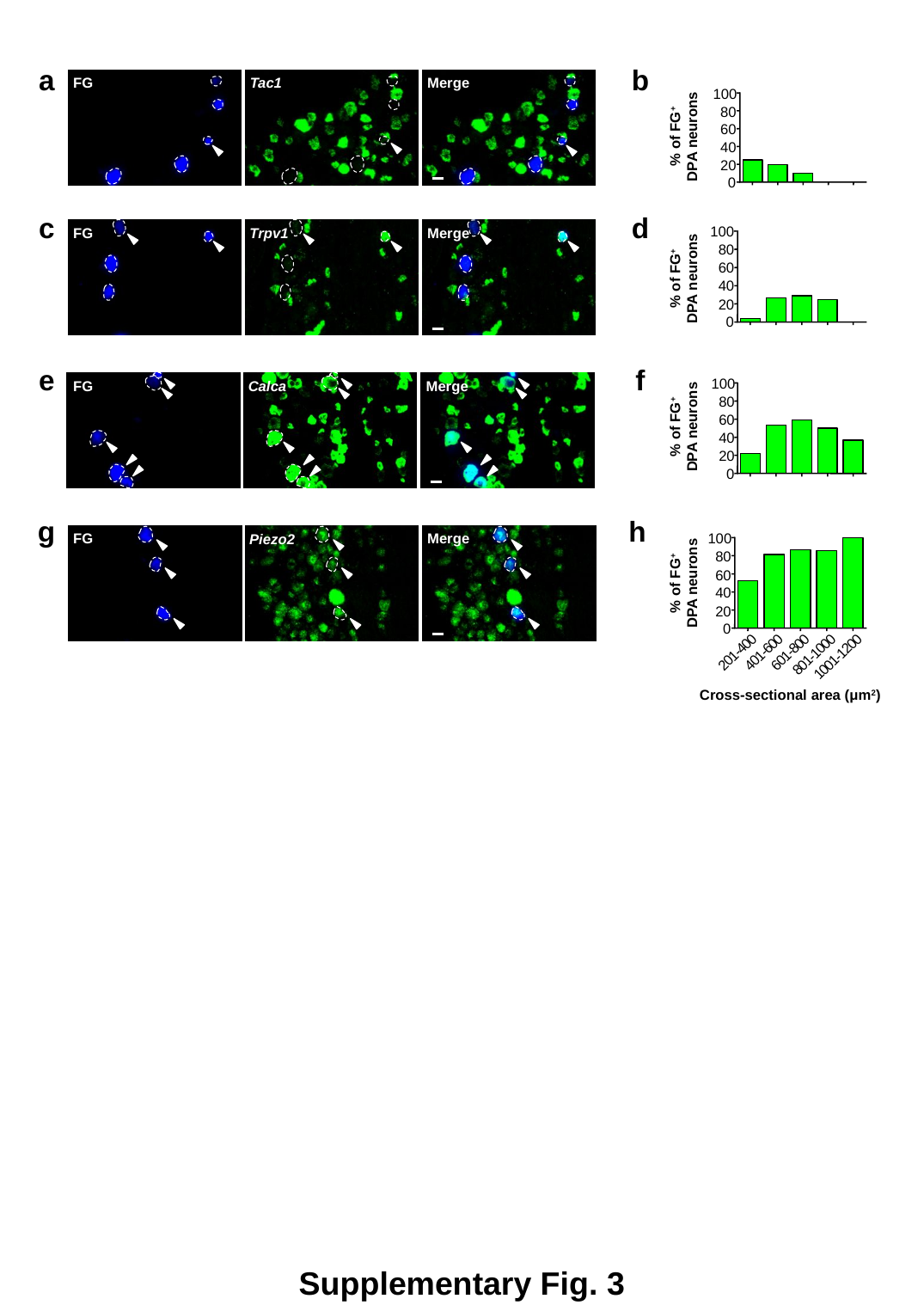

a
b
FG
Tac1
Merge
100
80
% of FG+
DPA neurons
60
40
20
0
c
d
FG
Trpv1
Merge
100
80
% of FG+
DPA neurons
60
40
20
0
e
f
100
80
% of FG+
DPA neurons
60
40
20
0
Calca
Merge
FG
g
h
FG
Merge
Piezo2
100
80
% of FG+
DPA neurons
60
40
20
0
0
0
0
0
0
0
0
0
0
0
4
6
8
0
2
-
-
-
1
1
1
1
1
-
-
0
0
0
1
1
2
4
6
0
0
8
0
1
Cross-sectional area (μm2)
Supplementary Fig. 3

## Slide 4
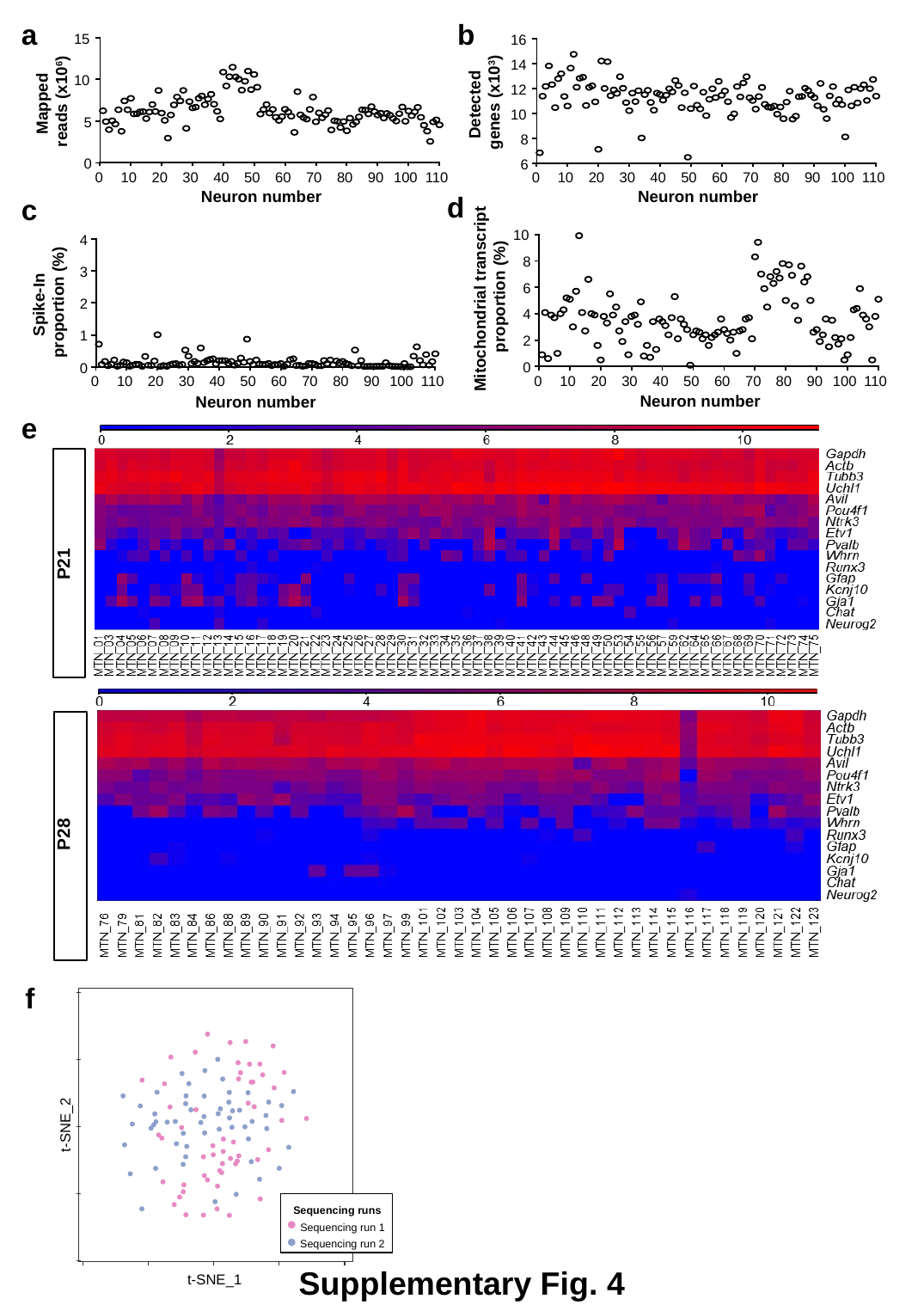

a
15
10
Mapped
reads (x106)
5
0
0
10
20
30
40
50
60
70
80
90
100
110
Neuron number
b
16
14
12
Detected
genes (x103)
10
8
6
0
10
20
30
40
50
60
70
80
90
100
110
Neuron number
d
10
8
6
Mitochondrial transcript
 proportion (%)
4
2
0
0
10
20
30
40
50
60
70
80
90
100
110
Neuron number
c
4
3
Spike-In
proportion (%)
2
1
0
0
10
20
30
40
50
60
70
80
90
100
110
Neuron number
e
P21
P28
f
t-SNE_2
t-SNE_1
Sequencing runs
Sequencing run 1
Sequencing run 2
Supplementary Fig. 4

## Slide 5
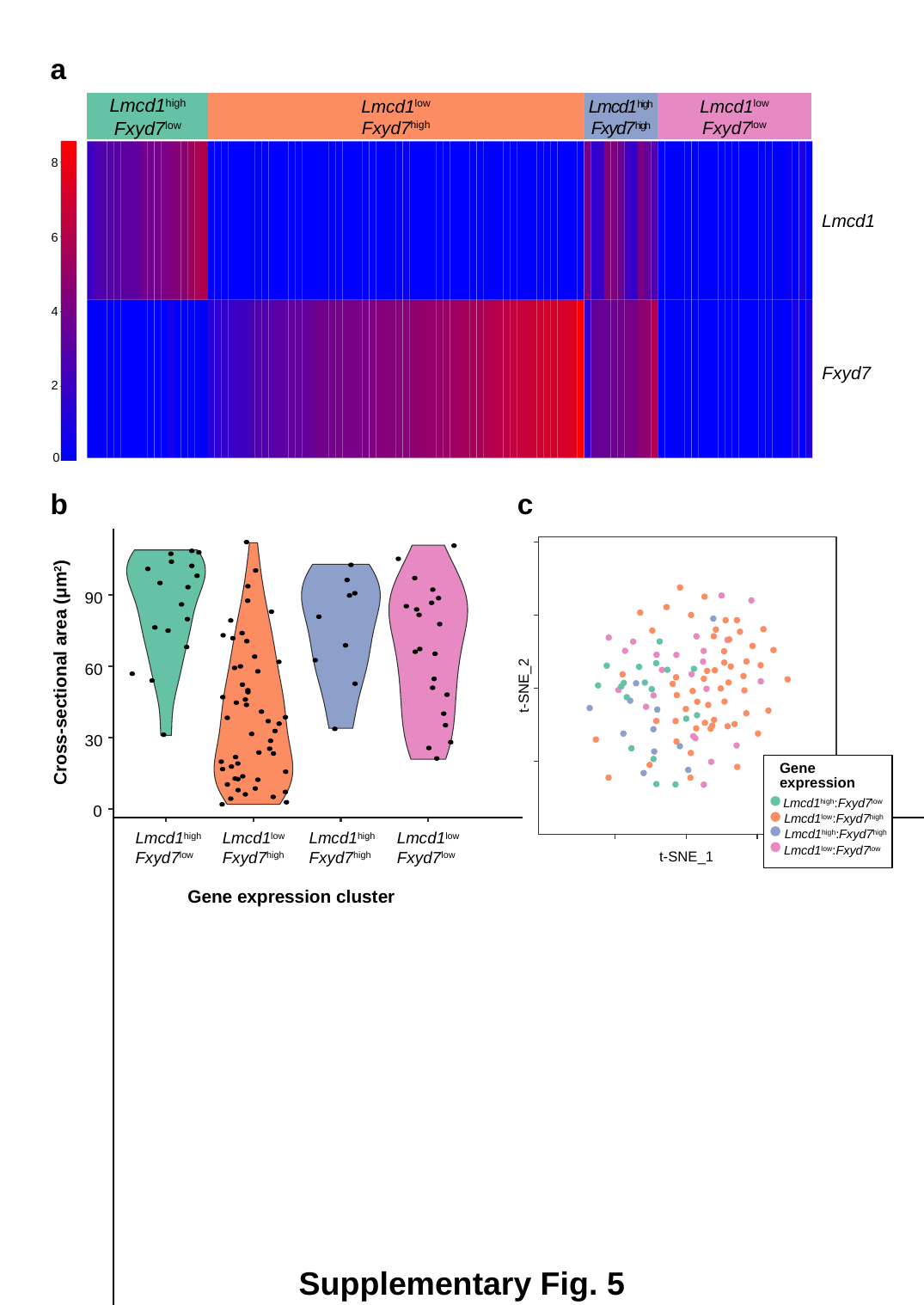

a
Lmcd1high
Fxyd7high
Lmcd1high
Fxyd7low
Lmcd1low
Fxyd7high
Lmcd1low
Fxyd7low
Lmcd1
Fxyd7
0
2
4
6
8
b
c
t-SNE_2
t-SNE_1
90
60
30
0
Lmcd1high
Fxyd7low
Lmcd1low
Fxyd7high
Lmcd1high
Fxyd7high
Lmcd1low
Fxyd7low
Cross-sectional area (μm2)
Gene expression cluster
Gene
expression
Lmcd1high:Fxyd7low
Lmcd1low:Fxyd7high
Lmcd1high:Fxyd7high
Lmcd1low:Fxyd7low
Supplementary Fig. 5

## Slide 6
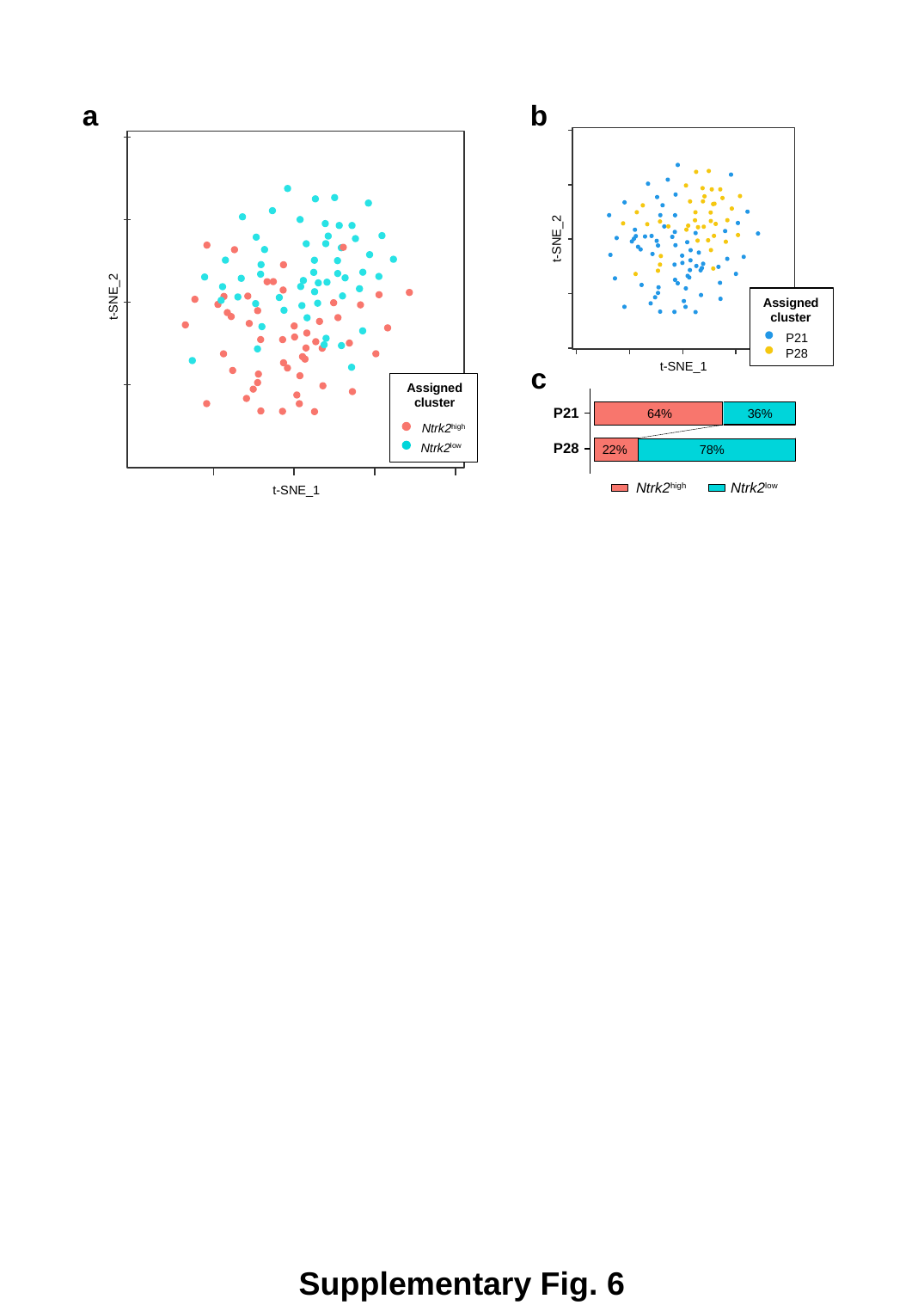

a
b
t-SNE_2
t-SNE_1
Assigned
 cluster
P21
P28
t-SNE_2
t-SNE_1
Assigned
cluster
Ntrk2high
Ntrk2low
c
P21
64%
36%
P28
22%
78%
Ntrk2high
Ntrk2low
Supplementary Fig. 6

## Slide 7
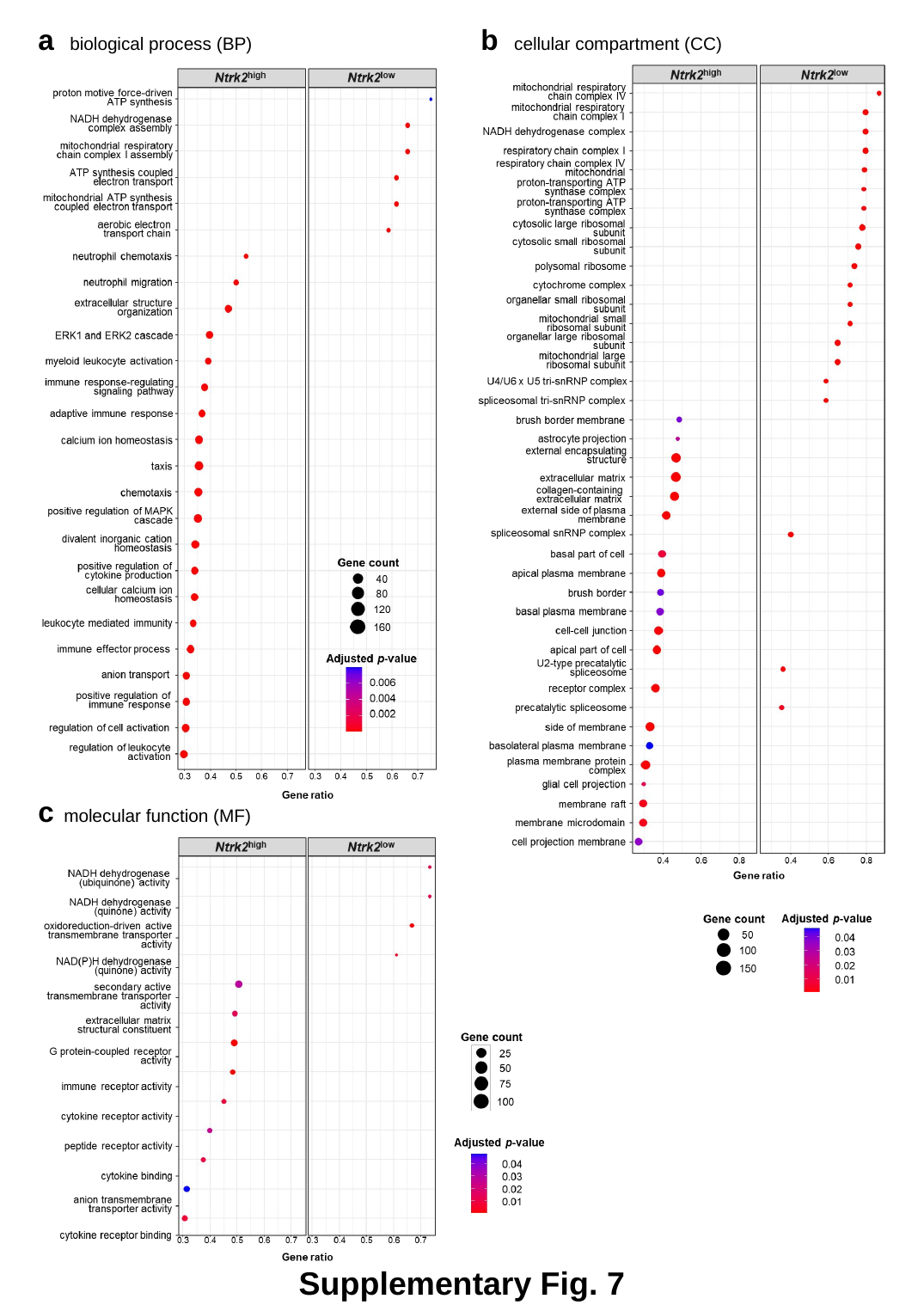

a biological process (BP)
b cellular compartment (CC)
c molecular function (MF)
Supplementary Fig. 7
